# Combining evidence, biomedical literature and statistical dependence: new insights for functional annotation of gene sets

**DOI:** 10.1186/1471-2105-7-241

**Published:** 2006-05-04

**Authors:** Marc Aubry, Annabelle Monnier, Celine Chicault, Marie de Tayrac, Marie-Dominique Galibert, Anita Burgun, Jean Mosser

**Affiliations:** 1 CNRS UMR 6061 Génétique et Développement, Université de Rennes 1, Groupe Oncogénomique, IFR140 GFAS, Faculté de Médecine, 2 avenue du Pr. Léon Bernard, CS 34317, 35043 Rennes Cedex, France; 2 OUEST-genopole^® ^Transcriptomic PF, IFR140 Génétique Fonctionnelle Agronomie et Santé, Université de Rennes 1, 35043 Rennes Cedex, France; 3 EA 3888 Modélisation Conceptuelle des Connaissances Biomédicales, Faculté de Médecine, Université de Rennes 1, 35043 Rennes Cedex, France

## Abstract

**Background:**

Large-scale genomic studies based on transcriptome technologies provide clusters of genes that need to be functionally annotated. The Gene Ontology (GO) implements a controlled vocabulary organised into three hierarchies: cellular components, molecular functions and biological processes. This terminology allows a coherent and consistent description of the knowledge about gene functions. The GO terms related to genes come primarily from semi-automatic annotations made by trained biologists (annotation based on evidence) or text-mining of the published scientific literature (literature profiling).

**Results:**

We report an original functional annotation method based on a combination of evidence and literature that overcomes the weaknesses and the limitations of each approach. It relies on the Gene Ontology Annotation database (GOA Human) and the PubGene biomedical literature index. We support these annotations with statistically associated GO terms and retrieve associative relations across the three GO hierarchies to emphasise the major pathways involved by a gene cluster. Both annotation methods and associative relations were quantitatively evaluated with a reference set of 7397 genes and a multi-cluster study of 14 clusters. We also validated the biological appropriateness of our hybrid method with the annotation of a single gene (cdc2) and that of a down-regulated cluster of 37 genes identified by a transcriptome study of an *in vitro *enterocyte differentiation model (CaCo-2 cells).

**Conclusion:**

The combination of both approaches is more informative than either separate approach: literature mining can enrich an annotation based only on evidence. Text-mining of the literature can also find valuable associated MEDLINE references that confirm the relevance of the annotation. Eventually, GO terms networks can be built with associative relations in order to highlight cooperative and competitive pathways and their connected molecular functions.

## Background

The numerous gene clusters identified thus far in molecular biology by high throughput analyses such as transcriptomic or proteomic technologies need to be understood according to the biological conditions under study. However, often only highly specialized individual biologists have an in-depth knowledge about a gene or gene product and therefore this knowledge is limited to relatively narrow research fields. The functional annotation of groups of gene products identified by genomic studies is a large challenge and new tools are needed to help in this task.

Ontologies are widely used in informatics and are now becoming important in bioinformatics. They can make the large amounts of biological knowledge found in textbooks and research papers generally accessible in a structured way [1]. Although the definition of an ontology can be very technical [2], it can be considered as a formalised area of knowledge, represented by facts (or concepts, or terms) and their logical connections (or relationships). The most important current uses of bio-ontologies are for representing knowledge in a way that is understandable by computers, for cross databases interoperability, and for annotating and analysing large-scale data. The Gene Ontology (GO) [3] is the *de facto *standard for formalising our knowledge about biological processes, molecular functions and cellular components, in three independent hierarchies [4]. It contains over 18,000 defined terms and the nodes within each hierarchy are connected by *is_a *or *part_of *relationships. As the defined terms can have more than one parent, the structure of this ontology is called a Directed Acyclic Graph (DAG). Furthermore, each GO term is associated with a unique identifier (GO ID) in order to allow a biological database to link to the GO and to ensure interoperability between different biological databases. These GO IDs are used in several biological databases – from almost 20 experimental organisms such as animals, plants, fungi, bacteria and viruses – to tag gene products and assign functions, biological roles and sub-cellular locations to them. Therefore, a user can identify the gene products associated with a specific GO term as well as all of the GO terms associated with this gene product by using an appropriate browser, such as *AmiGo *[5] or *GenNav *[6]. The investigation of gene function is therefore an important application of bio-ontologies and this has been extended to include the exploration of gene clusters. There are many dedicated analysis tools, such as *FatiGO *[7], *GoMiner *[8], *MAPPFinder *[9], *GOTree Machine *[10], *Onto-Tools *[11] or *GOToolBox *[12], that offer automated, practical and efficient solutions for retrieving GO terms associated to a gene cluster. Most are able to find statistically over-represented GO terms in a set when compared to a reference set – this can be the complete genome or the entire microarray used in the experiment [12,13].

Nevertheless, annotating genes with a controlled vocabulary is laborious and needs an expert to inspect carefully the literature associated with each gene to determine the appropriate terms. As our knowledge of biology increases, becomes more refined, and expands into new areas such a process will no longer be sufficient [14]. It is therefore obvious that the annotation databases are incomplete: the number of gene products and associated data are increasing faster than they can be annotated, and there are genes for which attributes are not yet well known and for which the literature has not yet been investigated by curators. It is thus undeniable that most of the information about gene functions is primarily contained in the records of the MEDLINE database, which is considered the richest and most accurate source of functional information related to genes [10,14,15]. However, this information is not easily understood by computers and is not easily interpretable on a large scale for both humans and computers. However, Natural Language Processing (NLP) methods allow gene-reference relationships to be identified within a scientific text, and many studies have been done to transfer named entity recognition systems to the biological domain. However, their success has been limited because of the highly dynamic nature of research and the complexity of entity names in the biological domain [16-18]. Only a few groups have tried to associate gene symbols with GO terms: MedMiner [19] and PubGene [20] can find gene-term associations based on co-occurrences although other methods have been considered, such as clustering [15], maximum entropy analysis [14] and keyword mapping [21].

We report here an original method for the functional annotation of gene clusters based on both evidence and literature profiles that aims to overcome the weaknesses and the limitations of each approach (annotation based on evidence and literature mining). We can functionally annotate a gene cluster by retrieving associated GO terms from two different sources of information. The first is an annotation database built on evidence: the Gene Ontology Annotation (GOA) database [22], and the second is a gene-term association database built on automated knowledge extraction from the biomedical literature: the *PubGene *index [23]. The *PubGene *method uses a probabilistic score to reflect the gene-term association strength. This score takes into account the frequencies of both gene and term in the 14 million article records of the database. We discard weak associations (*i.e*., score > 0.01) to improve the precision of the PubGene method. The two sets of GO terms are then merged and GO terms having statistically enriched gene numbers are identified to aid the biological interpretation of the cluster.

We evaluated the precision of each method and the overlap between them. We also evaluated the relevance of the bibliographic references associated with the gene cluster by the literature mining method. As we were seeking the biological meaning of a gene set, we focused on identifying metabolic pathways. This therefore limited the annotations to the Process hierarchy of GO.

Although subsumption (*is_a *relationship) and meronymy (*part_of *relationship) are the backbone of GO and make it a proper ontology in the computational sense, it however lacks associative relations within and especiallly across its three hierarchies. These relations would be very helpful and informative. For example, they could show that a certain molecular function is involved in a certain biological process and that a certain cellular component is the location of a certain biological process. We previously investigated three non-lexical approaches for identifying associative relations between GO terms [24]. We have used these dependences here to strengthen the previous annotations and to build a network of inter-dependent terms. This network highlights relationships that could exist between annotated pathways and functions.

This paper is organised as follows. The **Results **section describes how we compared evidence (GOA) and literature (PUB) using an exhaustive reference set of 7397 genes annotated by both methods. We then explain how we evaluated the contribution of statistical dependences (DEP) on the same reference set. The methodology was quantitatively evaluated in a multi-cluster analysis concerning 14 clusters chosen from 7 independent studies (Table 1). The biological consistency of our methodology was assessed for a minimal cluster of one single gene (cdc2) and for two clusters from a study by Bedrine-Ferran et al. [25] related to transcriptomic variations in human CaCo-2 cells used as an *in vitro *model of enterocyte differentiation (Up and Down clusters). A qualitative evaluation was also performed for two oncogenomic studies: a glioblastoma cluster (glioGBM) and a leukemia cluster (bcr-abl). The **Discussion **section describes the benefits and limitations of the evidence and literature annotations. We then comment on the major contributions of the bibliographic aspects of our method and that of the statistical dependence between GO terms. The **Methods **section details the technical and statistical aspects of our methodology.

## Result

### Reference gene set

The evidence annotation of the reference set provided 1625 Process terms whereas the literature annotation provided 3226. The two methods shared 1079 terms (24.9%). Although the reference set represented only 49.6% of the overall Process hierarchy of GO (8730 terms), we checked its relevance by evaluating its representativeness compared to all the GO Process terms available in the GOA Human database [22] and in the PubGene database. In both cases, the set covered more than 80% of the available terms and was therefore appropriate as a reference set.

#### Evidence codes (GOA)

In the evidence annotation, many gene-term associations were based on electronic inference: 38.5% of the terms retrieved in GOA were associated with the "Inferred from Electronic Annotation" evidence code (IEA) [26]. The remaining 61.5% corresponded to annotations made or reviewed by curators: 39% "Traceable Author Statement" (TAS), 10.8% "Non-traceable Author Statement" (NAS), 7.3% in the "Inferred from ..." family (IC: Curator, IDA: Direct Assay, IEP: Expression Pattern, IGI: Genetic Interaction, IMP: Mutant Phenotype, IPI: Physical Interaction and ISS: Structural Similarity), 3.4% "Not Recorded" (NR) and 1% "No biological Data available" (ND).

#### Probabilistic score (PubGene)

PubGene retrieved 269172 gene-term associations of which 38703 (14.4%) had a scored below 0.01. Among the 3236 Process terms associated with the set, 10 (0.3%) were obsolete and 3075 (95.3%) had a score below 0.01.

#### Number of genes per term

The generalised estimating equations (gee) showed that the annotation method did not affect the number of genes associated with a given term (estimated regression coefficient = 0.0035, standard error = 0.0893, p = 0.968). Terms with significantly enriched gene numbers could then be compared between methods.

#### Number of terms per gene

The literature annotation provided more than twice as many terms for a given gene than the evidence annotation (PUB 5,559 > GOA 2,485, Kruskal-Wallis χ^2 ^= 1886.863, df = 1, p-value < 2.2e-16).

#### Number of references per gene

There were many more references associated with one gene in the literature annotation than in the evidence annotation (PUB 73,520 > GOA 1,666, Kruskal-Wallis χ^2 ^= 4445.078, df = 1, p-value < 2.2e-16).

#### Depth (granularity)

We found no significant difference in the granularities of the two annotations (Kruskal-Wallis χ^2 ^= 1.5565, df = 1, p-value = 0.2122). The median depth was seven and was consistent with the overall granularity of the GO Process hierarchy.

**Figure 1:**
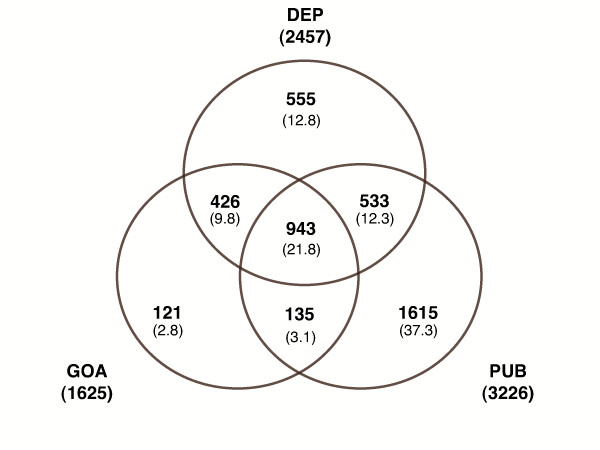
**Venn diagram for the reference set**. Number of GO Process terms in each category (overall percentage in brackets). Evidence annotation (GOA), Literature annotation (PUB) and Associative relations (DEP).

As the two annotation methods have similar representativeness, number of genes per term and granularity, we added the associative relations to the two previous term sets. Figure 1 shows the overlap between the three sources of GO terms. The core of common terms remained large with 21.8% of the terms. In the evidence annotation, 84.2% of the terms were also retrieved by dependence. By contrast, the literature method was more specific, with only 45.7% of the terms being found by dependence and 50.1% being original terms.

#### Path quality index (PQI)

**Figure 2:**
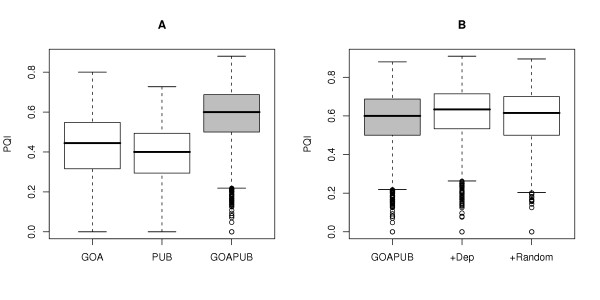
**Reference set**. (A) Boxplots of the PQIs for the Evidence (GOA), Literature (PUB) and combination of both (GOAPUB). (B) Boxplots of the PQIs for the evidence and literature terms (GOAPUB), for the same set enriched with the associative relations (+Dep.) or a random term set of the same size (+Random).

For a GO term, the Path Quality Index (PQI) is a measure of its relative number of annotated parents and children terms. PQIs for the combination of both evidence and literature were significantly different from evidence alone (Kruskal-Wallis χ^2 ^= 922.5441, df = 1, p-value < 2.2e-16) or literature alone (Kruskal-Wallis χ^2 ^= 2302.722, df = 1, p-value < 2.2e-16, Figure 2A). The PQIs naturally increased when dependent or random terms were added to the combination of evidence and literature annotations. This increase was significantly different (Kruskal-Wallis χ^2 ^= 40.065, df = 1, p-value = 2.457e-10) for the dependent set versus the random set (Figure 2B).

### Minimal cluster: a single gene (cdc2)

**Figure 3:**
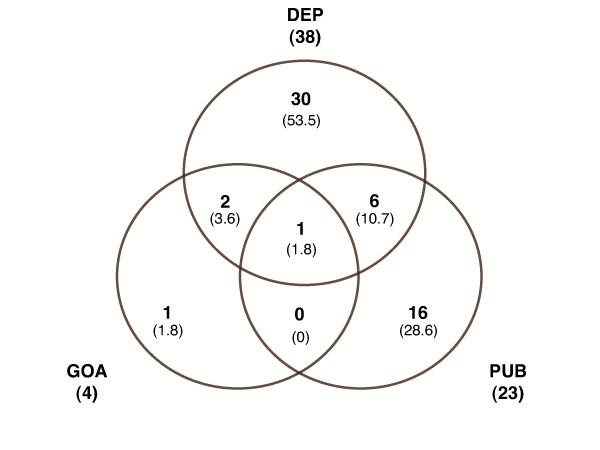
**Venn diagram for cdc2**. Number of GO Process terms in each category (overall percentage in brackets). Evidence annotation (GOA), Literature annotation (PUB) and Associative relations (DEP).

**Figure 4:**
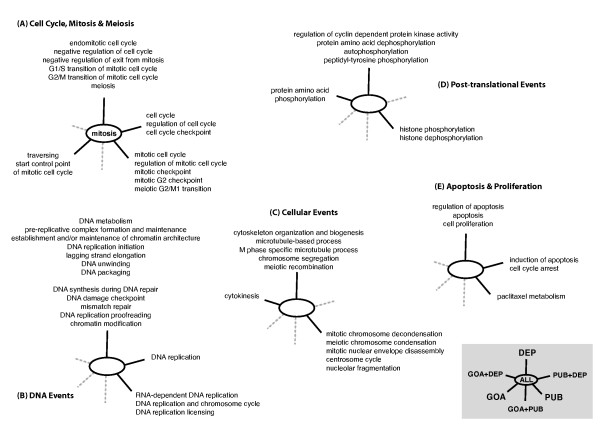
**Venn categories for the functional annotation of the cdc2 gene (Process hierarchy)**. Annotations: GOA (Evidence), PUB (Literature), DEP (Associative relations) and overlaps between them (GOA+PUB, GOA+DEP, PUB+DEP, ALL = GOA+PUB+DEP).

The number of terms obtained by each method for cdc2 in the Process hierarchy is shown in Figure 3. We limited the literature profile to significant terms (p <= 0.01) and discarded associative relations having a PQI of zero. The terms in each Venn category are graphically presented in Figure 4. The terms were sorted into five categories for clarity: (A) cell cycle, mitotis and meiosis, (B) DNA events occuring during the cell cycle, including generic DNA replication and controls, (C) cellular events occuring during the cell cycle, mainly related to the cytoskeleton, (D) post-translational events and kinase-associated processing, (E) apoptosis and proliferation.

Evidence annotation retrieved only four Process terms and, with the exception of "traversing start control point of mitotic cell cycle" (GO:0007089), all were found by literature profiling and/or associative relations. The literature annotation retrieved 154 Process terms, 23 of which had scores below 0.01. These significant terms were associated with 266 MEDLINE references. A systematic reading of the title and abstract of these references showed that these were relevant for the associations brought out and related all the important steps of the cdc2 characterisation (discovery of the cell cycle mutants for the yeast, biochemical purification of the Mitosis Phase Factor (MPF) in several species, descriptions of the various substrates and inhibitors of cdc2, etc.). Furthermore, half the references provided by the literature annotation were less than 5 years old.

**Figure 5:**
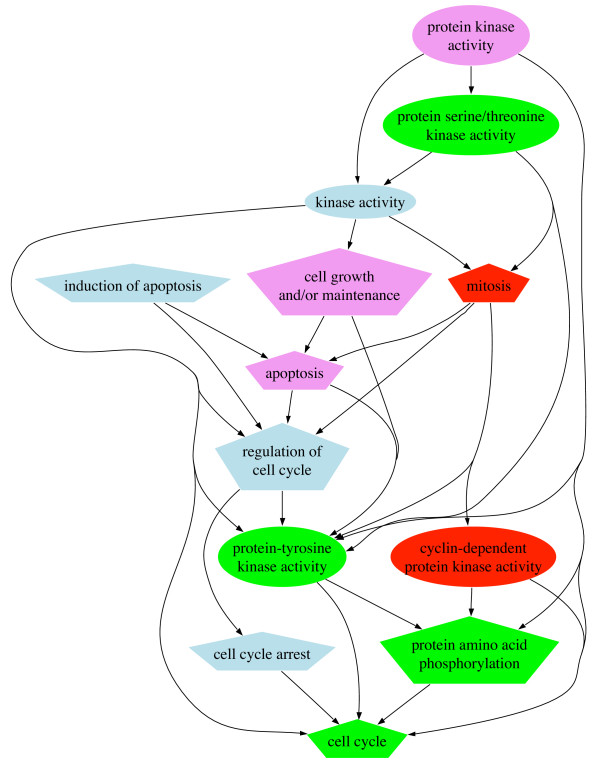
**A selective part of the network of inter-dependent terms for cdc2. **Shape: ellipse = Function, polygon = Process. Annotations: GOA (Evidence), PUB (Literature) and DEP (Associative relations). Color: Venn categories, red = GOA+PUB+DEP, green = GOA+DEP, lightblue = PUB+DEP, violet = DEP. Arrows indicate the way the dependence was found in our associative database.

Terms from the evidence and literature annotations were also associated with 153 terms in the associative relation database. Only 38 of these had a non-zero PQI. A selective part of the network of terms associated by dependence is presented in Figure 5.

### Down cluster

**Figure 6:**
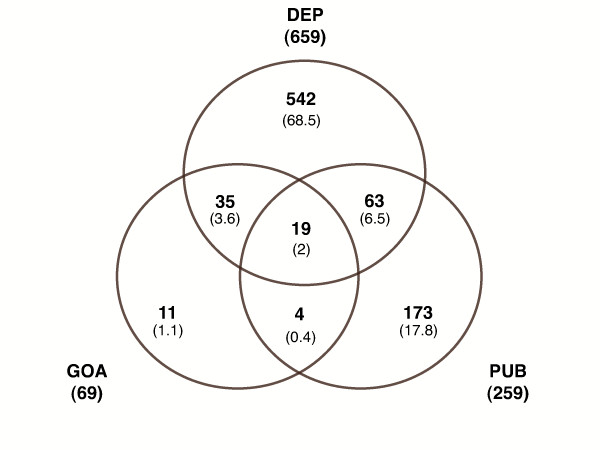
**Venn diagram for the Down cluster**. Number of GO Process terms in each category (overall percentage in brackets). Evidence annotation (GOA), Literature annotation (PUB) and Associative relations (DEP).

A Venn diagram for the 37 down-regulated genes during CaCo-2 cells differentiation is shown in Figure 6. We limited the literature profile to significant terms (p <= 0.01) and discarded associative relations having a PQI of zero.

#### Evidence

As for the reference set, genes from the Down cluster were primarily annotated with three evidence codes: IEA (47.1%), TAS (33.3%) and NAS (12.6%). TAS, NAS and IDA evidence codes were associated with 28 MEDLINE references. Manual inspection of the 87 gene-term associations confirmed the accuracy of the evidence annotation and the robustness of the inference methods used in building annotation databases. Less than 2% of the terms were unexploitable. These were either misassociated, for example "perception of sound" (GO:0007605) with ITM2B, or not very biologically informative, for example "biological_process unknown" (GO:0000004) for TRIP6.

#### Literature

The Down cluster was annotated with 259 significant GO terms associated with 3377 MEDLINE references. Manual inspection of all the 626 significant gene-term associations retrieved by PubGene showed that 81.5% had a direct link between the gene and the term, *e.g*., "copper ion transport" (GO:0006825) with ATP7B, "DNA replication initiation" (GO:0006270) with MCM3, "chromatin silencing" (GO:0006342) and "DNA packaging" (GO:0006323) with CBX1, or "ornithine catabolism" (GO:0006593) and "putrescine catabolism" (GO:0009447) with ODC1. There were very few false positives associations (1.2%). The remaining 17.3% of the associations were correct but imprecise. The gene symbol and the term were both found effectively in the title/abstract but there was either: (i) no biological relationship between them, for example, ATP7B associated with "mRNA metabolism" (GO:0016071) in a study of mRNA expression levels (and thus transcription) of ATP7B itself [27], or (ii) the biological relationship between them was indirect. For example, the relationship between "cell cycle checkpoint" (GO:000075) and EIF3S2 (eukaryotic translation initiation factor 3, subunit 2) from a study by Humphrey and Enoch [28] on sum1+ (suppressor of uncontrolled mitosis) is indirect because this protein presents a "striking sequence similarity" with EIF3S2. Similarly, "regulation of cell cycle" is indirectly related to TOP2A as shown by Pasion et al. [29] in a study demonstrating the "negative regulation of TOP2A mRNA during the cell cycle".

#### Combination of evidence and literature

**Figure 7:**
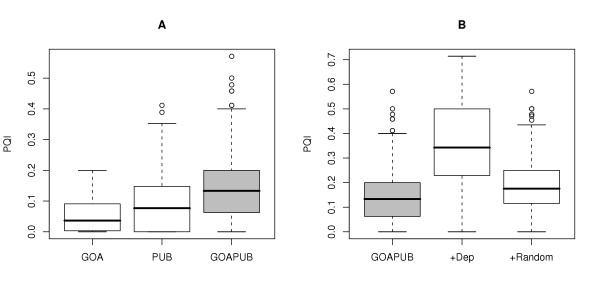
**Down cluster**. (A) Boxplots of the PQIs for the Evidence (GOA), Literature (PUB) and combination of both (GOAPUB). (B) Boxplots of the PQIs for the evidence and literature terms (GOAPUB), for the same set enriched with the associative relations (+Dep.) or a random term set of the same size (+Random).

PQIs for the combination of both methods were significantly different from evidence alone (Kruskal-Wallis χ^2 ^= 48.9203, df = 1, p-value = 2.666e-12) or literature alone (Kruskal-Wallis χ^2 ^= 41.2014, df = 1, p-value = 1.373e-10) (Figure 7A).

#### Terms with significantly enriched gene numbers

**Figure 8:**
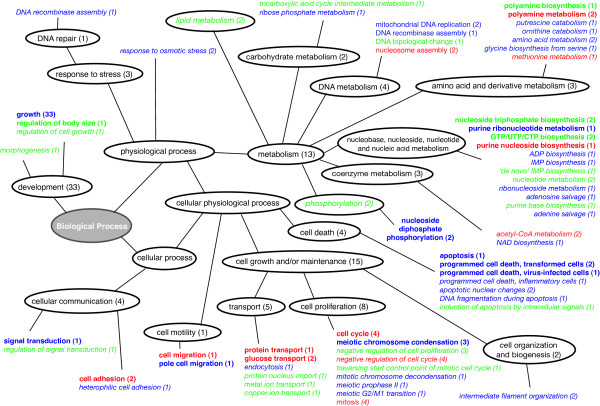
**Enriched GO terms for the Down cluster**. Terms highly enriched (p ≤ = 0.01) in bold and terms enriched (p ≤ = 0.05) in italic. Number of associated genes in brackets. Colors: red = terms found in GOA and PubGene, green = terms only found in GOA, blue = terms only found in PubGene.

Enriched GO terms for the Down cluster are shown in Figure 8. At a threshold of 0.01, the cluster is characterised by processes also described in the Bedrine-Ferran study: cell cycle, transport, signal transduction, nucleic acid and polyamine metabolism. These metabolic pathways underlie the proliferative state of undifferentiated CaCo-2 cells. Among the additional pathways retrieved by our method, two were strongly annotated and relevant: apoptosis and growth. At a threshold of 0.05, the enrichment either supplies additional terms to the annotated pathes, specifically cell death, cell proliferation and nucleic acid metabolism, or identifies new functional areas, such as DNA metabolism. See Additional file 1 for the complete annotation of the Down cluster.

#### Associative relations

**Figure 9:**
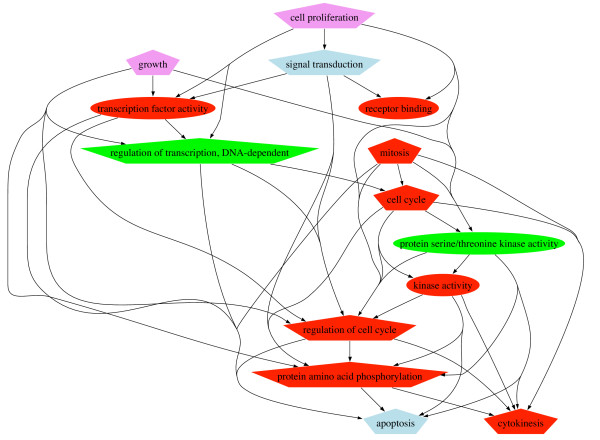
**A selective part of the inter-dependent terms network for the Down cluster**. Shape: ellipse = function, polygon = process. Annotations: GOA (Evidence), PUB (Literature) and DEP (Associative relations). Color: Venn categories, red = GOA+PUB+DEP, green = GOA+DEP, lightblue = PUB+DEP, violet = DEP. Arrows indicate the way the dependence was found in our associative database.

The addition of dependent or random terms to the evidence and literature annotations naturally increased the PQIs. This increase was significantly different (Kruskal-Wallis χ^2 ^= 255.7346, df = 1, p-value < 2.2e-16) for the dependent set versus the random set (Figure 7B). We found a higher, although not unreasonable, proportion of bad associations (about 10%) from the systematic inspection of all the 256 gene-term-term associations retrieved in the associative relations database for the Down cluster. We identified three different types of errors: (i) Term-term misassociations, for example, the process "positive regulation of smooth muscle contraction" (GO:0045987) and the function "G-protein-coupled receptor binding" (GO:0001664); (ii) term-gene misassociations, such as the process "response to pheromone" (GO:0019236) for HSPA9B; and (iii) gene-term-term misassociations, for example, the function "calcium ion binding" (GO:0005509) and the processes "synaptic transmission" (GO:0007268) and "neuropeptide signaling pathway" (GO:0007218) for ANXA5. Relevant associations comprised within hierarchy (WH) dependences (35%) and across hierarchies (AH) dependences (65%). A selective part of the inter-dependent terms network for the Down cluster is shown in Figure 9.

### Multi-cluster analysis

The 14 clusters used in the multi-cluster analysis are presented in Table 1. The number of terms in each Venn categories for all these clusters can be found in Table 2. Results concerning the PQIs comparisons can be found in Table 3 and Additional files 2, 3, 4, 5, 6, 7 and 8 for the corresponding boxplots. The annotation of the glioGBM cluster is consistent with the conclusions of Tso et al. [50], reflecting characteristics of hyper-proliferation, hypervascularity, and apoptotic resistance in glioblastoma clusters. A DAG of the enriched annotated terms associated with at least 4 genes is provided in the Additional file 9. For the bcr-abl cluster, the retrieved processes are in full accordance with the pathogenesis of the BCR/ABL Acute Lymphoblastic Leukemia (ALL) [51]. Indeed, the high proliferation rate of the blast cells is highlighted by numerous cell cycle processes including cytokinesis and chromosome segregation, but also by the activation of the MAPKKK cascade and its links with cell cycle checkpoints and anti-apoptosis processes that lead to cell survival [52]. It correlates secondly with the angiogenesis process, linking the BCR/ABL fusion protein to VEGF (vascular endo-thelial growth factor) gene expression [53] which is a hallmark of tumor aggressiveness. A DAG of the enriched annotated terms associated with a least 4 genes is provided in the Additional file 10.

## Discussion

### Evidence

Our study shows that GOA provides high-quality GO annotations with a 98% precision despite there being a large number of electronically inferred gene-term associations (about 50%). Bad associations are mostly indirect rather than entirely false. For example, the "perception of sound" (GO:0007605) associated with ITM2B comes from a spkw2go mapping [30] as this gene was implicated in causing deafness. These results are consistent with a recent evaluation carried out by Camon et al. [31]. The obvious limitation of such an annotation method is that manual processing capability could rapidly become overloaded and would no longer be able to deal with the increasing amount of scientific data. Therefore, semi-automatic methods are often used to speed up the curation process. Although these semi-automatic methods are primarily used to assist biologist curators [31] and have proven to be useful [32], they are very rarely used as automatic annotation tools. This is especially true when based on text mining of MEDLINE references [33].

### Literature

Text mining of biomedical literature combined with probabilistic scoring of the gene-term associations is also a powerful annotation technique. For a given gene set, it retrieved more terms per gene than evidence annotation and with a similar precision. Although common terms highlighted the major pathways, supplementary terms were a valuable source of information for reinforcing those pathways, by adding parent, sibling and child nodes. For example, in the annotation of cdc2 (Figure 4), the literature profile retrieved "regulation of cell cycle" (GO:0000074) and "cell cycle checkpoint" (GO:0000075). These are respectively the parent and sibling of "traversing start control point of mitotic cell cycle" (GO:0007089) found by evidence. Likewise, the literature term "histone phosphorylation" (GO:0016572) is the child of the evidence term "protein amino acid phosphorylation" (GO:0006468). Literature terms are thus valuable for improving the coherence of the annotation, but they also retrieve recent biologically characterised path. For example, in the annotation of the Down cluster, "paclitaxel metabolism" (GO:0042616) and "paclitaxel biosynthesis" (GO:0042617) were associated with three gene products: CDKN1A, CDC2 and TOP2A. Although the inhibitory effect of paclitaxel (taxol) on *cdc2 *was identified ten years ago, its action on CDKN1A and TOP2A was only recently characterised [34].

### Bibliographic insights

Scientific literature is the optimal resource for validating a functional annotation. However, GOA provides few MEDLINE references to support its annotation. Despite there being abundant literature on cdc2 only one article was retrieved: a general study by Laronga et al. [35] on cyclin-dependent kinases in which cdc2 was only used for an *in vitro *kinase assay. Moreover, the annotators linked this article to the Cellular Component "nucleus" (GO:0005634) whereas it would be better associated to "negative regulation of cell cycle" (GO:0045786) and "negative regulation of cyclin-dependent protein kinase activity" (GO:0045736). Likewise, HMGA2 was TAS-associated with "development" (GO:0007275) [36] whereas we would expect the GO terms to describe this protein as an architectural factor involved in adipogenesis – "fat cell differentiation" (GO:0045444) for example – and mesenchyme differentiation, as suggested in the article abstract and in more recent studies [37]. Most of the references found in GOA are used to justify the annotation and that is their purpose. Therefore, they are often referent references, such as the discovery of the gene product or its first characterisation, and are less informative when searching for recent advances in the field. Given the limitations of the manual processing, there is no reference at all for many genes in GOA Human (*e,g*., ODC1, CBX1, LAMB1).

The significant increase in the associated MEDLINE references in the literature annotation corroborates the enrichment of the GO terms. The considerably higher number of associated references and their accuracy (up to 90% precision) make PubGene an excellent bibliographic tool for validating the biological interpretation of a cluster. In the Down cluster, PubGene was able to retrieve very informative references highlighting the main biological implications of one gene. For example, the study by Chen et al. [38] was associated with eight GO terms for ODC1 and described its involvement in the polyamine and derivative (ornithine and putrescine) metabolisms. The literature approach can also retrieve gene sub-clusters based on their common references. These sub-clusters can group genes by family, for example, KRT8 and KRT18 co-annotated in four references and NME1 and NME2 co-annotated in two references, or by shared processes, for example, HSPCA with HMGB1 [39], CDKN1A with TAGLN [40], and CDKN1A with cdc2 and TOP2A [35].

As expected, the primary source of errors found in the literature approach was linked to the ambiguity of the gene symbols: CBX1 was associated with "secretion" (GO:0046903) whereas the abstract of Dodic et al. [41] referred to CBX (carbenoxo-lone), SON (SON DNA binding protein) was confused with SON (SupraOptic Nucleus) in the paper by Eguchi et al. [42] and was therefore inappropriately associated with "vasopressin secretion" (GO:0030103). Other errors may be more difficult to resolve: ANXA5 was annotated with "prostanoid biosynthesis" (GO:0046457) in a reference to a study by McGinty et al. [43] on Cyclooxygenase-2 (Cox-2) – an enzyme responsible for catalyzing the committed step in prostanoid biosynthesis – in which ANXA5 was only stained to assess the trophic withdrawal apoptosis level in pheochromocytoma cells. These problems stress the importance of the formalism provided by the HUGO Gene Nomenclature Committee (HGNC) [44].

### Statistical dependence

Our data strongly suggest that networks of statistically inter-dependent GO terms highlight the leading features of a gene or gene cluster: a synthetic and simplified interpretation of its annotation. Most of the main processes identified in the functional annotation of the Down cluster (Figure 8: cell cycle and cell proliferation, growth, cellular communication, cell death) were also part of the inter-dependent terms network (Figure 9: cell cycle and cell proliferation, growth, signal transduction, apoptosis). This network also emphasised the most specific mechanisms involved in this cluster: cellular proliferation and growth is correlated with transcription phenomena required for the cell cycle and mitosis. The regulation of these processes involves specific kinase activities and can be either positive (cytokinesis) or negative (apoptosis).

The associative relations primarily provided dependences within and especially across the GO hierarchies and linked functions to processes. With statistical dependence, biologically meaningful relations were found: (i) between GO terms across hierarchies, such as the "signal transduction" (GO:0007165) process with the "receptor binding" (GO:0005102) function in the Down cluster annotation, or the "mitosis" (GO:0007067) process and the "cyclin-dependent protein kinase activity" (GO:0004693) function in the cdc2 annotation; and (ii) between GO terms belonging to different sub-DAGs of the same hierarchy, such as the "regulation of cell cycle" (GO:0000074) and "apoptosis" (GO:0006915) processes in both the Down cluster and cdc2 annotations.

### Future directions

We have presented here an application of the associative relations to the functional interpretation of experimental results. We deliberately restricted their contribution to reinforce the evidence or literature annotated pathways and to identify between annotated terms the relationships across hierarchies. This improvement needs to be evaluated in terms of the precision and specificity of each non-lexical approach and the term-term associations could also be filtered with respect to their similarity coefficient.

The biological interpretation of a gene cluster will surely be facilitated by the identification of the GO sub-DAGs having a high number of annotated nodes. Each term in the gene cluster annotation has a PQI that measures its annotation degree: its relative number of co-annotated kinship terms. Using the distribution of the PQI within the DAG, it is therefore possible to identify statistically over-annotated sub-DAGs – possibly biological pathways – linked to a specific biological condition. Nevertheless, this measure needs to be normalised in order to be independent from the size of the gene cluster and, consequently, from the number of GO terms in the annotation.

We used the associative relations to identify possible interactions between processes and functions but this method is general to GO and not specific to a gene cluster. At least two other approaches could be explored at the cluster level. The first and most obvious one is to link terms that share one or more genes (co-annotated genes). These terms are likely to be the enriched terms from the annotation. Such approach can yet be elusive as most of the terms are only annotated with one gene. A second approach is to link sub-DAGs with high PQI terms (co-annotated terms) if we consider the PQI as a quantification of a sub-DAG (pathway) relevance for the gene cluster (biological condition).

## Conclusion

Despite their obvious differences, semi-automatic annotation based on evidence and literature mining combined with statistical scoring of the gene-term associations are both efficient methods for associating relevant GO Process terms to a gene cluster. The significantly higher PQIs obtained using a combination of both methods is an indication of their synergy: they do not contain the same information. We achieved a more robust and complete annotation by combining the coherence of GOA with PubGene's exploratory and bibliographic qualities. Eventually, GO terms networks can be built with associative relations in order to highlight cooperative and competitive pathways and their connected molecular functions. Our methodology is an effort to improve the actual situation which is clearly suboptimal. It is, however, not demonstrated to what precise degree this improvement goes. This remains to be determined but is outside the scope of the present paper.

## Methods

### Sources of GO terms

#### GOA: annotation based on evidence (GOA Human)

The GOA database aims to provide high-quality supplementary GO annotation to proteins in the UniProt (SWISS-PROT/TrEMBL) databases. Most of the GOA content comes from the manual curation of scientific literature, with semi-automatic and electronic techniques being used to support the annotation process. Therefore, an evidence code assesses the reliability of the gene-term association. These codes are established by the GO Consortium [26] and range from electronically inferred to experimental evidence. As all the associations in GOA are gene-term associations, the only way to rank terms in clusters is to use the number of genes associated with each term. We limited our study to the annotation of human genes and gene products in the GOA Human database (93136 associations for 22720 distinct proteins and 10085 MEDLINE references in the December 2004 release).

#### PUB: annotation based on literature (PubGene)

PubGene is a web-based database of gene-gene and gene-term associations based on co-occurrences in biomedical literature. It provides a full-scale literature network for 25,000 human genes extracted from the titles and abstracts of over 14 million article records from the MEDLINE citation database of the National Library of Medecine (NLM). The method assumes that if two genes are mentioned in the same MEDLINE record there should be an underlying biological relationship. Genes are linked to terms from the Gene Ontology and a probabilistic score is computed that reflects the gene-term association strength which can be used to assess the relevance of each individual term. The computation of this probabilistic score assumes that occurrences of the gene and the term are independent. Therefore, a binomial formula can be used to estimate the probability of finding the gene and the term together in an article based on their respective frequencies in the whole database. Assuming a normal distribution, the expected number of articles mentioning the gene and the term is then compared to the number of times they actually occur together (see [46] for details). In clusters, the reliability of each term is a multiplication of its probabilistic scores. The literature annotation was carried out with the 2.4 release of the PubGene database (December 2004). Obsolete terms were replaced by updated ones if present in the term_definition table of the GO database (*i.e*., specified in the term_comment attribute) and obsolete terms with no updated term were discarded from the literature annotation. Terms being poorly associated with the gene cluster (*i.e*., probabilistic score greater than 0.01) were also discarded.

#### DEP: statistical dependences (associative relations)

The lack of representation in GO of the relations existing among functions, processes and components severely limits the power of reasoning based on GO. In a previous work, we investigated three non-lexical approaches for identifying associative relations between GO terms: the vector space model, statistical analysis of co-occurrences and association rules mining [24]. Here, we used the associative relations database we built (term-term associations) to strengthen the previous annotations: we queried this database with evidence terms and significant literature terms and retrieved a list of dependent terms (gene-term-term associations). The link between the Process "oxygen transport" (GO:0015671) and the Component "hemoglobin complex" (GO:0005833) is an example of such associative relation.

### Gene sets

#### Reference gene set

We built a reference set of 7397 human genes that we used for a quantitative evaluation of our approach and to identify the statistically significant enriched *GO *terms in the functional annotation of a given experimental gene set. We downloaded the Human Gene Nomenclature Database [44] from the *HUGO *Gene Nomenclature Committee on the 9 th of December 2004. It contained 20056 different mapped LocusLink IDs. We preferred mapped LocusLink IDs because these are subjected to a peer-review process. We annotated 13505 IDs in *GOA Human *and found that 10969 of them presented either an approved symbol or an alias or older symbol that could be used to query the *PubGene *database. These queries gave 7397 effective annotations. The *GOA *Human annotation was then restricted to this subset to carry out comparison.

#### Minimal cluster: a single gene (cdc2)

We wanted to determine what the method was able to retrieve for a minimal cluster, that is, for a single gene. We chose the cell division cycle 2 (*cdc2*) product, involved in the G2/M transition of the cell cycle [47], because its functions and regulations are well-known and fully documented.

#### Multi-cluster analysis

The methodology was quantitatively evaluated in a multi-cluster analysis concerning 14 clusters chosen from 7 independent studies. Detailed informations on these clusters can be found in Table 1. The qualitative evaluation and the biological relevance of our methodology was assessed with the two clusters from the study by Be-drine-Ferran et al. [25] related to transcriptomic variations in human CaCo-2 cells used as an *in vitro *model of enterocyte differentiation. These clusters are differentially expressed genes through the differentiation process: 30 up-regulated genes (Up cluster) and 37 down-regulated genes (Down cluster). Evaluations for both Up and Down clusters were quantitatively and qualitatively similar. We will therefore only detail here the results obtained for the down-regulated cluster (Down cluster). See Additional file 11 for LocusLink IDs, symbols, aliases and names of these down-regulated genes.

### Identification of GO terms with enriched gene numbers

When annotating a gene set with an hypothetical biological meaning the challenge is to find the *GO *terms that best characterise this set. These terms will be among those relevant to a high number of genes. We used the hypergeometric distribution to identify statistically significant enrichments (see [11] for a comparison of statistical methods). As a reference set, we used here the 7397 gene set built for evaluating our method.

### Terms attributes

The various parameters measured for each term and used to evaluate the contribution of each annotation method, compare them and bring forward their specificity are called attributes.

#### Attributes related to the methods

We measured, for each GO term, and for each annotation method, the number of occurrences and the number of associated genes. We carried out statistical analyses only on the number of genes per term because these two variables were strongly correlated (r = 0.997, p < 0.001). For the terms only found by both methods, we tested the number of genes per term against the annotation method (*i.e*., evidence annotation versus literature annotation). We carried out analyses with a generalized estimating equation (gee) model to estimate parameters for correlated data, assuming a Poisson error and a log-link function. We used the R package 'geepack' [48].

Evidence and literature verbosities were compared using a Kruskal-Wallis rank sum test on the number of GO terms per gene. Likewise, we used the Kruskal-Wallis rank sum test to compare the number of MEDLINE references per gene and to assess the bibliographic wealth in the evidence and literature methods.

#### Attributes related to the DAG

A Directed Acyclic Graph (DAG) is a hierarchy in which a node can have multiple parents and children. The highest node, the one having no parents is called the root node and the deepest nodes, those with no children, are the leaves. Thus, a node can be characterised by its position within the DAG. The depth or granularity of the node is its minimum distance from the root node [49]. We compared the granularities of the evidence and literature methods using the Kruskal-Wallis rank sum test. A GO term is thus part of a sub-DAG that includes all its parents in every path up to the root node and all its children and their descendants down to the leaves of the DAG. A term will be relevant if it has an enriched gene number. However, it will also be interesting if many of its parents and children are annotated. For a term, the Path Quality Index (PQI) is a measure of its relative number of annotated parents and children nodes: PQI = (N_Pa _+ N_Ca_)/N, where N is the total number of parents and children nodes in the sub-DAG, N_Pa _is the number of annotated parent nodes and N_Ca _is the number of annotated child nodes. We used the PQI to compare the evidence and literature annotations to a combination of both annotations. We also used it to evaluate the global relevance of the GO terms found only by dependence. In this case, we calculated the PQIs for the combination of the evidence and literature and compared it to the PQIs obtained after the addition of: (i) the terms found only by dependence, and (ii) a random term set of equal size. We compared PQIs using the Kruskal-Wallis Rank Sum Test with a Bonferroni correction for multiple comparisons. We eventually used the PQI to filter out the associative relations and to limit them to reinforcing the annotation: dependent terms with a zero PQI (*i.e*., terms with no annotated term in their sub-DAG) were discarded.

### Biological relevance

#### Evidence annotation

We carried out a systematic inspection of each gene-term association retrieved from the GOA Human database. Associations were sorted into three categories depending on their relevance: good associations in which the GO term was directly linked to the gene product, bad associations in which the GO term was misassociated with the gene product or non-informative, and doubtful associations in which the GO term could be indirectly linked to the gene product (*e.g*., a molecular mechanism implied by or associated with the gene activity but not the gene activity itself) or, inferred by a sequence similarity, etc.

#### Literature profile

We manually inspected each of the MEDLINE references retrieved by PubGene. We read the title and abstract and classified the relevance of each gene-term association into the same three categories as used for the evidence annotation.

#### Associative relations

For the Down cluster, we manually investigated each associative relation found only by dependence and having a non-zero PQI. We determined whether the associated terms were biologically related or not and wether the association between the dependent term and the gene was appropriate. Associative relations were also sorted into Within Hierarchy (WH) relations and Across Hierarchies (AH) relations.

### Databases and tools

The version of GO used throughout this study is the February 2005 monthly release, available from the GO website. DAG graphical representations were achieved using dot v1.10 and Graphviz 1.13(v16). All other graphics and statistical analyses were done using the R language version 2.1.0.

## Authors' contributions

All computational tasks and statistical analyses were carried out by MA. The biological relevance of the method was primarily evaluated by AM and assisted by CC, JM and MA. Expertises on glioblastomas and leukemias were respectively supplied by MdT and MG. AB and JM supervised this study and contributed to continuous discussions about its shortcomings. All authors have read and approved the final manuscript.

## Supplementary Material

Additional File 1 GO terms associated with the Down cluster: Venn category, GO ID, Name, GOA Human annotation with evidence codes and genes, PubGene annotation with significant scores and genes. Click here for file

Additional File 2 Up cluster. (A) Boxplots of the PQIs for the Evidence (GOA), Literature (PUB) and combination of both (GOAPUB). (B) Boxplots of the PQIs for the evidence and literature terms (GOAPUB), for the same set enriched with the associative relations (+Dep.) or a random term set of the same size (+Random). Click here for file

Additional File 3 Glioblastomas clusters. (A) Boxplots of the PQIs for the Evidence (GOA), Literature (PUB) and combination of both (GOAPUB). (B) Boxplots of the PQIs for the evidence and literature terms (GOAPUB), for the same set enriched with the associative relations (+Dep.) or a random term set of the same size (+Random). Click here for file

Additional File 4 Acute Lymphocyte Leukemias (ALL) clusters. (A) Boxplots of the PQIs for the Evidence (GOA), Literature (PUB) and combination of both (GOA-PUB). (B) Boxplots of the PQIs for the evidence and literature terms (GOAPUB), for the same set enriched with the associative relations (+Dep.) or a random term set of the same size (+Random). Click here for file

Additional File 5 Circadian cluster. (A) Boxplots of the PQIs for the Evidence (GOA), Literature (PUB) and combination of both (GOAPUB). (B) Boxplots of the PQIs for the evidence and literature terms (GOAPUB), for the same set enriched with the associative relations (+Dep.) or a random term set of the same size (+Random). Click here for file

Additional File 6 Lung cluster. (A) Boxplots of the PQIs for the Evidence (GOA), Literature (PUB) and combination of both (GOAPUB). (B) Boxplots of the PQIs for the evidence and literature terms (GOAPUB), for the same set enriched with the associative relations (+Dep.) or a random term set of the same size (+Random). Click here for file

Additional File 7 Retina clusters. (A) Boxplots of the PQIs for the Evidence (GOA), Literature (PUB) and combination of both (GOAPUB). (B) Boxplots of the PQIs for the evidence and literature terms (GOAPUB), for the same set enriched with the associative relations (+Dep.) or a random term set of the same size (+Random). Click here for file

Additional File 8 Alzheimer's disease cluster. (A) Boxplots of the PQIs for the Evidence (GOA), Literature (PUB) and combination of both (GOAPUB). (B) Boxplots of the PQIs for the evidence and literature terms (GOAPUB), for the same set enriched with the associative relations (+Dep.) or a random term set of the same size (+Random). Click here for file

Additional File 9 Enriched GO Process terms (p < = 0.05) associated with at least 4 genes in the glioGBM cluster. Color: red = terms found in GOA and PubGene, green = terms only found in GOA, blue = terms only found in PubGene. Shape: rectangle = significantly enriched annotated terms (p < = 0.05) ; ellipse = non-significantly enri-ched annotated terms (p > 0.05). Click here for file

Additional File 10 Enriched GO Process terms associated with at least 4 genes in the bcr-abl cluster. Color: red = terms found in GOA and PubGene, green = terms only found in GOA, blue = terms only found in PubGene. Shape: rectangle = significantly enriched annotated terms (p < = 0.05) ; ellipse = non-significantly enriched annotated terms (p > 0.05). Click here for file

Additional File 11 LocusLink IDs, symbols, aliases and names of the down regulated genes. Click here for file

## Figures and Tables

**Table 1:** Clusters used in the multi-cluster analysis.

Name	Size	Tissue	Reference
*Iron metabolism:*			
Down	37	Intestine	Bedrine-Ferran et al., *Genomics *(2004) [25]
Up	28		

*Glioblastomas:*			
glioGBM	15	Glia	Tso et al., *Cancer Res*. (2006) [50]
glioPRIM	58		
glioSEC	21		

*Circadian Rhythms:*			
circ	56	Heart & liver	Storch et al., *Nature *(2002) [54]

*Human fetal lung:*			
lung	17	Lung	Wade et al., *Am. J. Respir. Cell Mol. Biol*. (2006) [55]

*Human retinal pigment epithelium:*			
arpe19	9	Retina	Cai and Del Priore, *Mol. Vision *(2006) [56]
rpe	35		

*Alzeimers disease:*			
alzheimer	167	Hippocampus	Blalock et al.,*Proc. Natl. Acad. Sci*. (2004) [57]

*Acute lymphoblastic leukemias:*			
bcr-abl	35	Bone marrow	Yeoh et al., *Cancer Cell *(2002) [58]
hyperdiploid	38		
novel	37		
tel-aml1	34		

**Table 2:** Number of GO terms in each Venn categories for the multi-cluster analysis.

	Venn categories^†^						
	
Clusters	GOA	GOA+DEP	GOA+PUB	ALL	PUB+DEP	PUB	DEP
*Iron metabolism:*							
Down	11	35	4	19	63	173	542
Up	11	20	3	12	28	163	301

*Glioblastomas:*							
glioGBM	17	12	2	6	20	144	255
glioPRIM	16	41	7	8	56	219	410
glioSEC	6	19	2	13	23	107	241

*Circadian Rhythms:*							
circ	11	38	10	20	55	191	342

*Human fetal lung:*							
lung	10	21	2	3	10	88	247

*Human retinal pigment epithelium:*							
arpe19	3	12	0	2	10	31	180
rpe	10	37	9	9	35	177	419

*Alzeimers disease:*							
alzheimer	33	69	14	56	182	497	596

*Acute lymphoblastic leukemias:*							
bcr-abl	13	38	5	12	54	163	383
hyperdiploid	13	32	7	11	37	186	347
novel	9	21	1	9	26	119	319
tel-aml1	11	37	4	7	37	153	389

† Venn categories: GOA (Evidence), PUB (Literature), DEP (Associative relations) and overlaps between them (GOA+PUB, GOA+DEP, PUB+DEP, ALL = GOA+PUB+DEP).

**Table 3:** Comparison of the PQIs (Kruskal-Wallis Rank Sum Tests). See Additional files 2, Additional files 3, Additional files 4, Additional files 5, Additional files 6, Additional files 7 and 8 for the corresponding boxplots.

	Null hypotheses^†^					
	
	GOAPUB ≠ GOA		GOAPUB ≠ PUB		+Dep. ≠ +Random	
Clusters	χ2	p-value^‡^	χ2	p-value^‡^	χ2	p-value^‡^
*Iron metabolism:*						
Down	48.9203	***	41.2014	***	255.7346	***
Up	27.1614	***	41.1756	***	73.0033	***

*Glioblastomas:*						
glioGBM	29.7468	***	1.1743	ns	85.9769	***
glioPRIM	62.7100	***	90.2077	***	97.2004	***
glioSEC	0.6282	ns	1.5421	ns	76.2122	***

*Circadian Rhythms:*						
circ	53.1073	***	26.2437	***	126.8363	***

*Human fetal lung:*						
lung	19.0615	***	36.1988	***	16.3595	***

*Human retinal pigment epithelium:*						
arpe19	3.999	*	9.5254	*	42.7507	***
rpe	21.2666	***	30.0108	***	105.1405	***

*Alzeimers disease:*						
alzheimer	121.7634	***	151.0647	***	14.2819	***

*Acute lymphoblastic leukemias:*						
bcr-abl	41.6444	***	44.5055	***	137.2054	***
hyperdiploid	14.0618	***	22.3200	***	137.0784	***
novel	21.1505	***	91.1497	***	22.9347	***
tel-aml1	15.5670	***	6.5539	ns	118.602	***

† Null hypotheses: comparison of the PQIs between Evidence (GOA) and a combination of Evidence and Literature (GOAPUB ≠ GOA). Comparison of the PQIs between Literature (PUB) and a combination of Evidence and Literature (GOAPUB ≠ PUB). Comparison of the PQIs between the combination of Evidence and Literature enriched with the associative relations (+Dep.) or a random term set of the same size (+Random).

‡ p-value: ≤ 0.01 (***) ; ≤ 0.05 (*) ; non-significant (ns).
