# Supplementary figures and images for: Combining evidence, biomedical literature and statistical dependence: new insights for functional annotation of gene sets
Source: BMC Bioinformatics. 2006 May 4;7:241. doi: 10.1186/1471-2105-7-241 (PMC1482722; doi:10.1186/1471-2105-7-241)

**Up (A)**

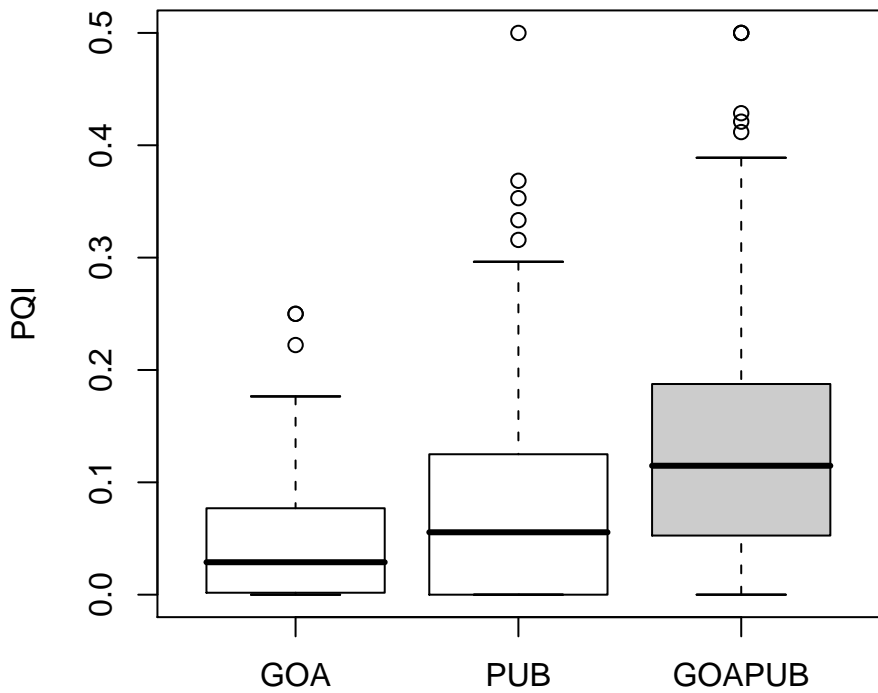

**Up (B)**

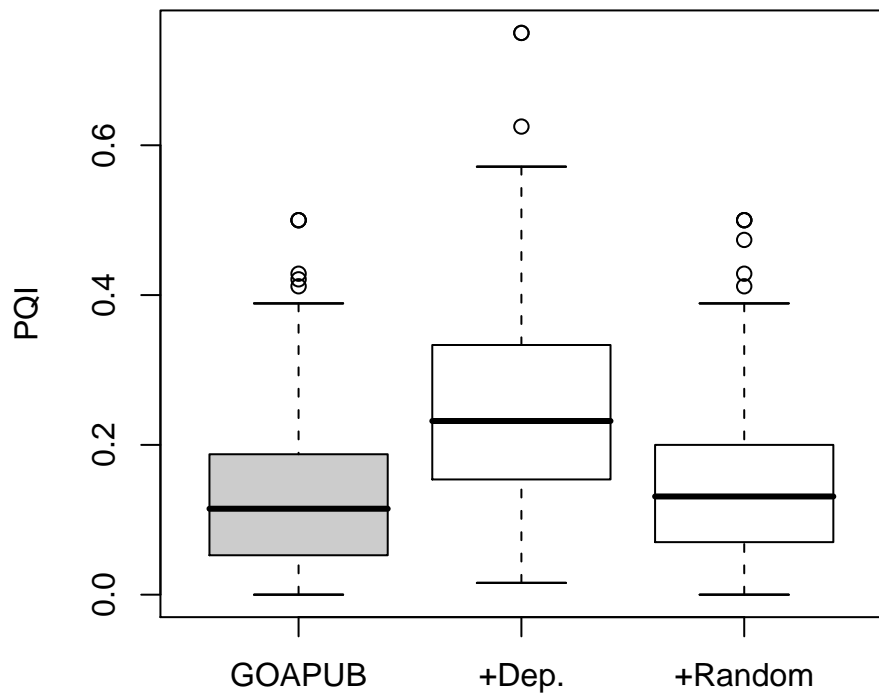

Supplement: Additional File 2 — Up cluster. (A) Boxplots of the PQIs for the Evidence (GOA), Literature (PUB) and combination of both (GOAPUB). (B) Boxplots of the PQIs for the evidence and literature terms (GOAPUB), for the same set enriched with the associative relations (+Dep.) or a random term set of the same size (+Random). [file 1471-2105-7-241-S2.pdf]

**glioGBM (A)**

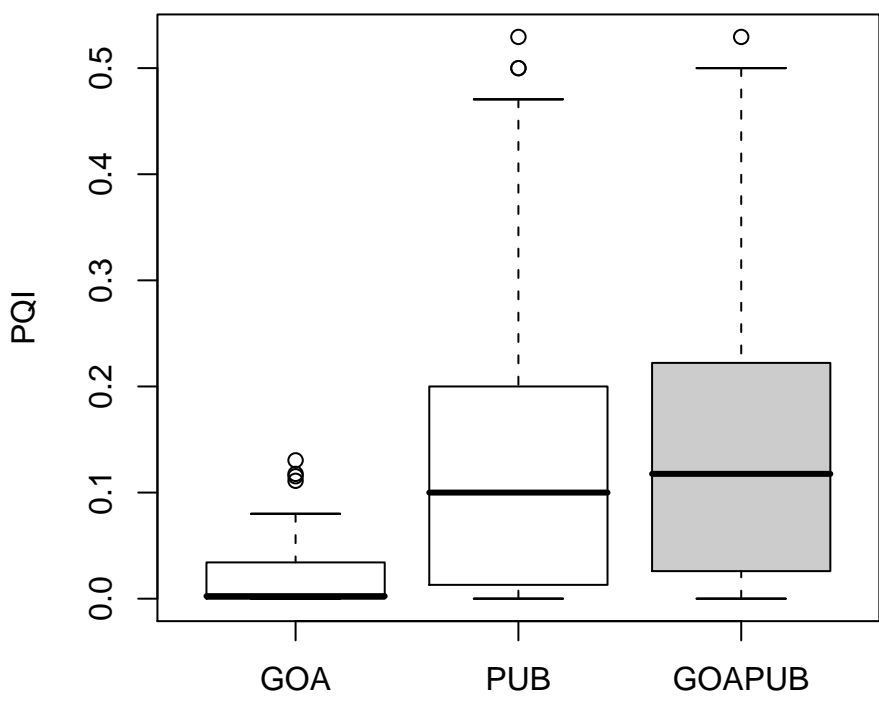

**glioGBM (B)**

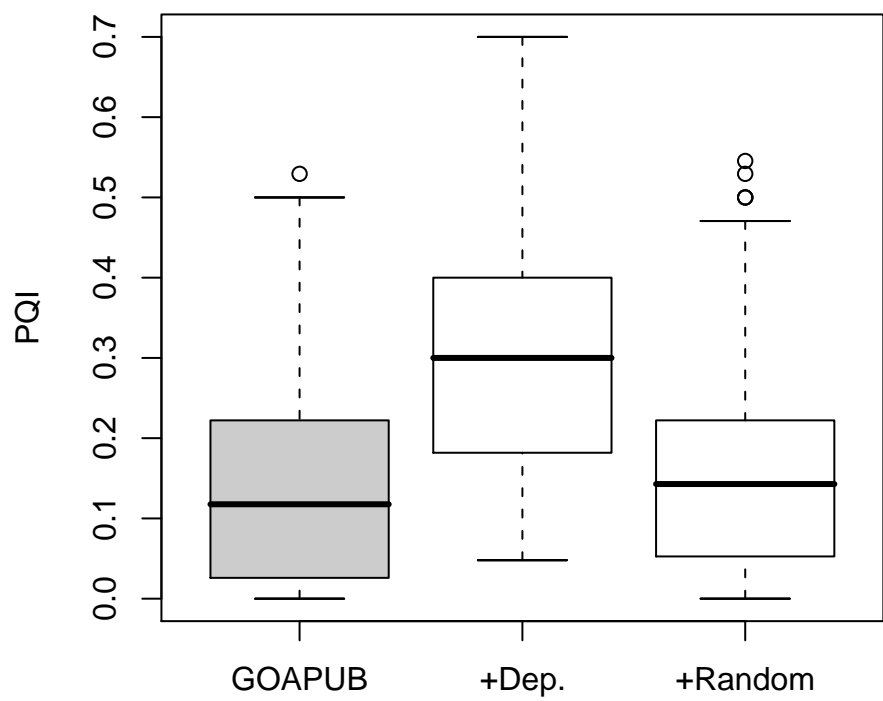

**glioPRIM (A)**

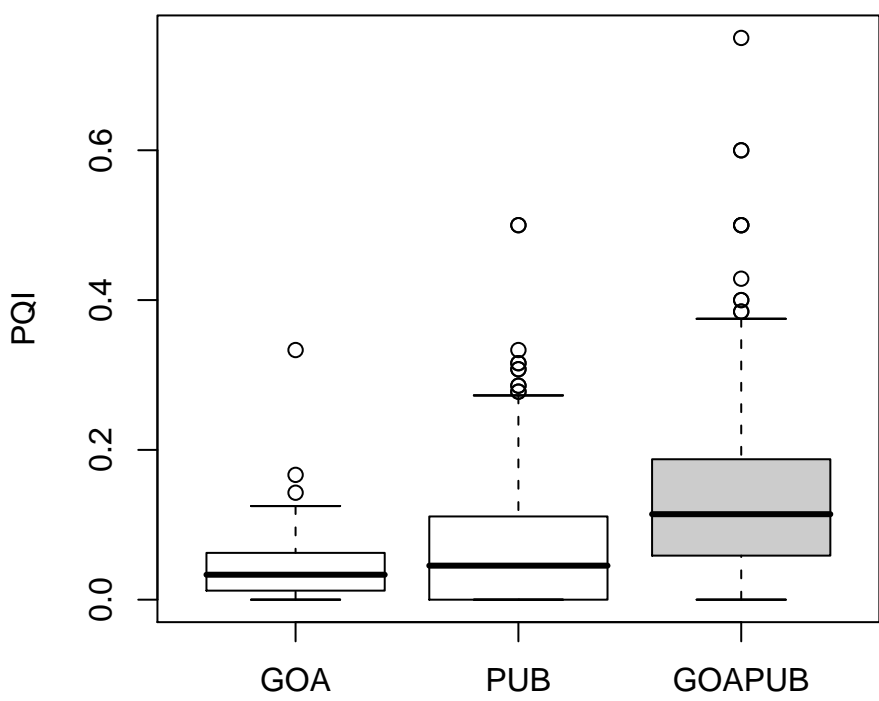

**glioPRIM (B)**

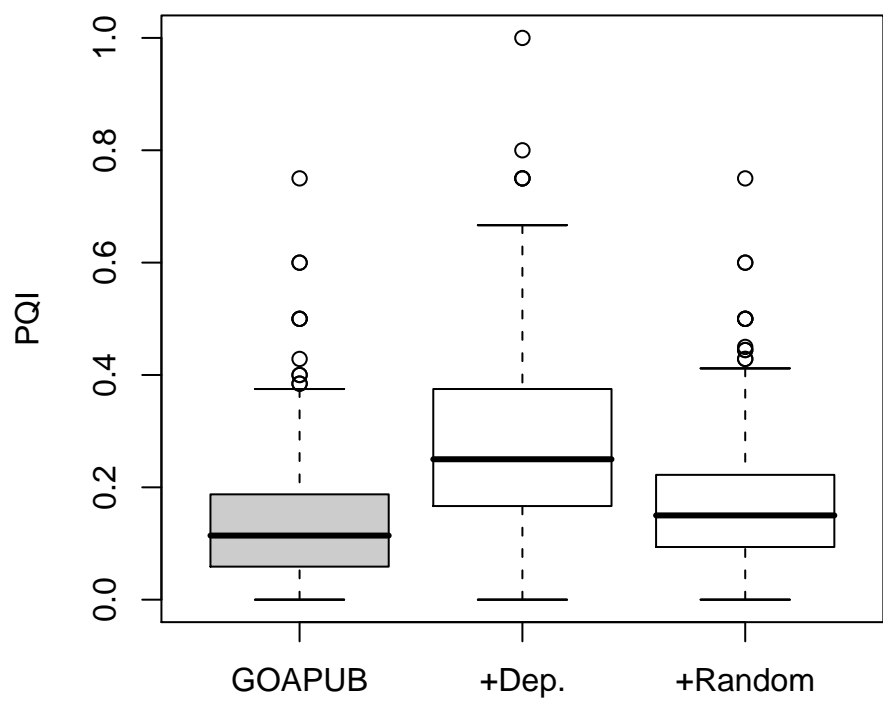

**glioSEC (A)**

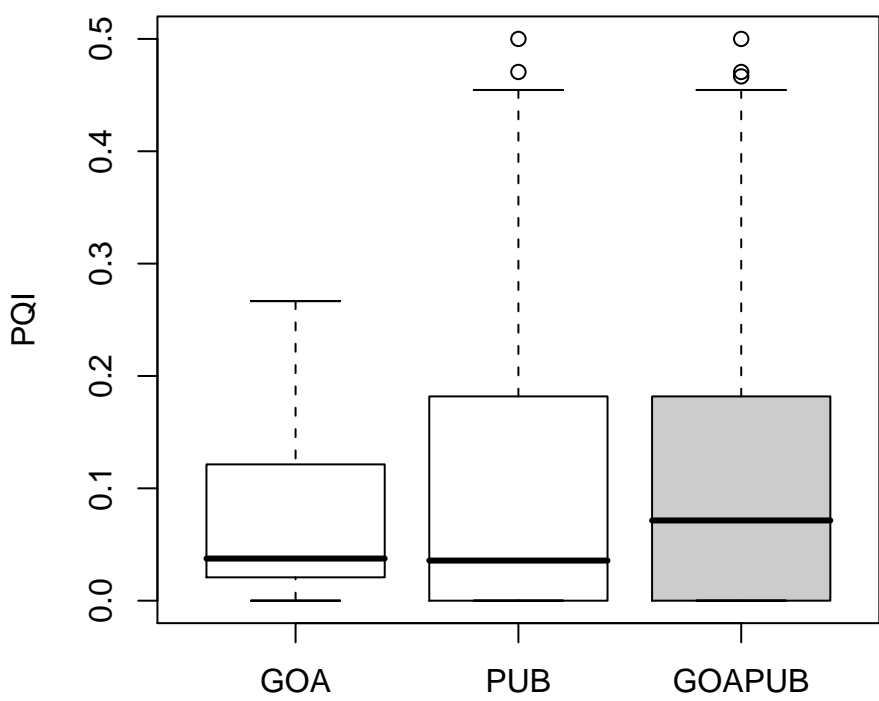

**glioSEC (B)**

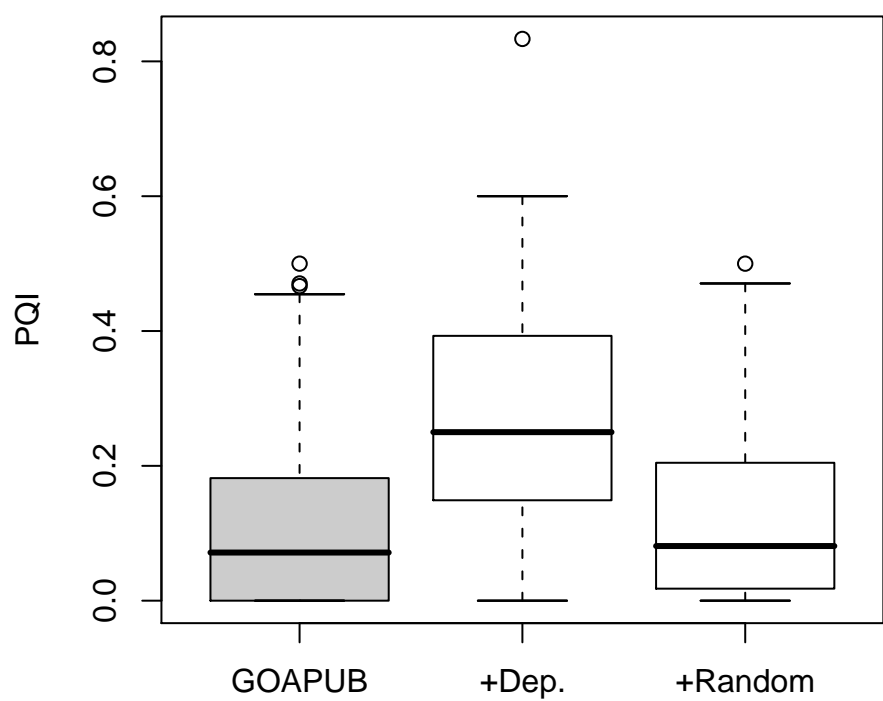

Supplement: Additional File 3 — Glioblastomas clusters. (A) Boxplots of the PQIs for the Evidence (GOA), Literature (PUB) and combination of both (GOAPUB). (B) Boxplots of the PQIs for the evidence and literature terms (GOAPUB), for the same set enriched with the associative relations (+Dep.) or a random term set of the same size (+Random). [file 1471-2105-7-241-S3.pdf]

**bcr-abl (A)**

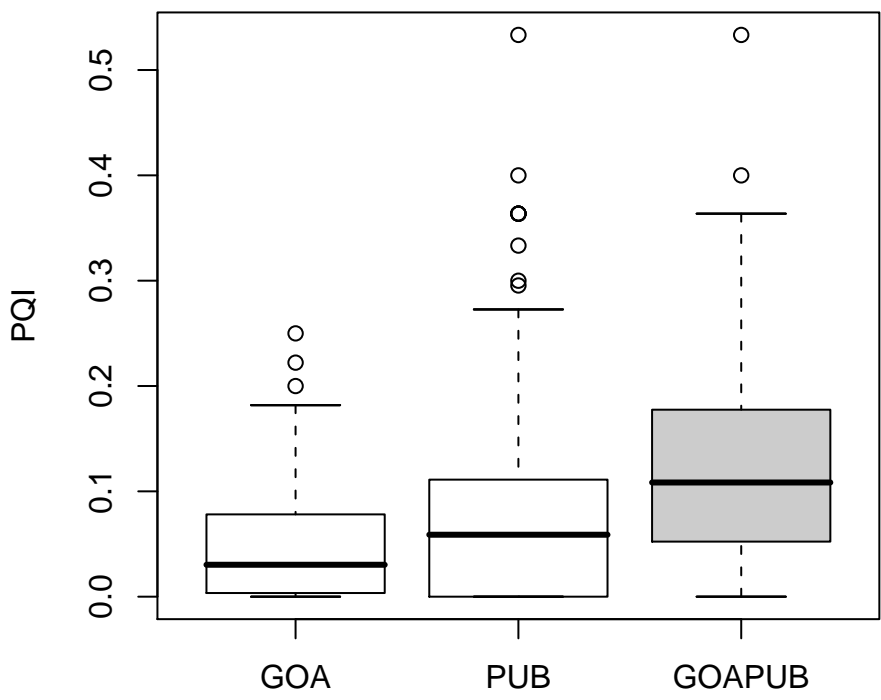

**bcr-abl (B)**

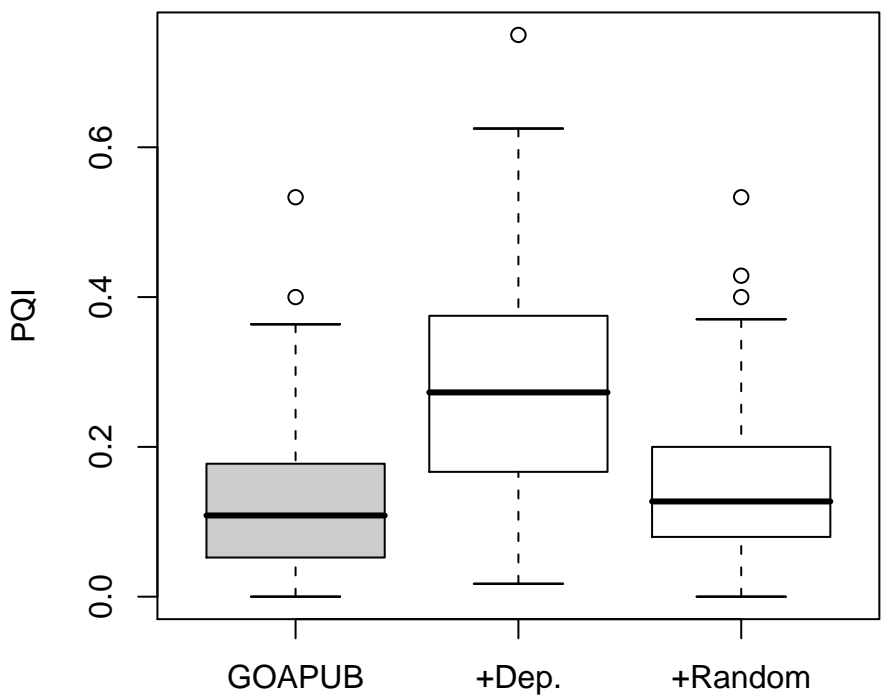

**hyperdiploid (A)**

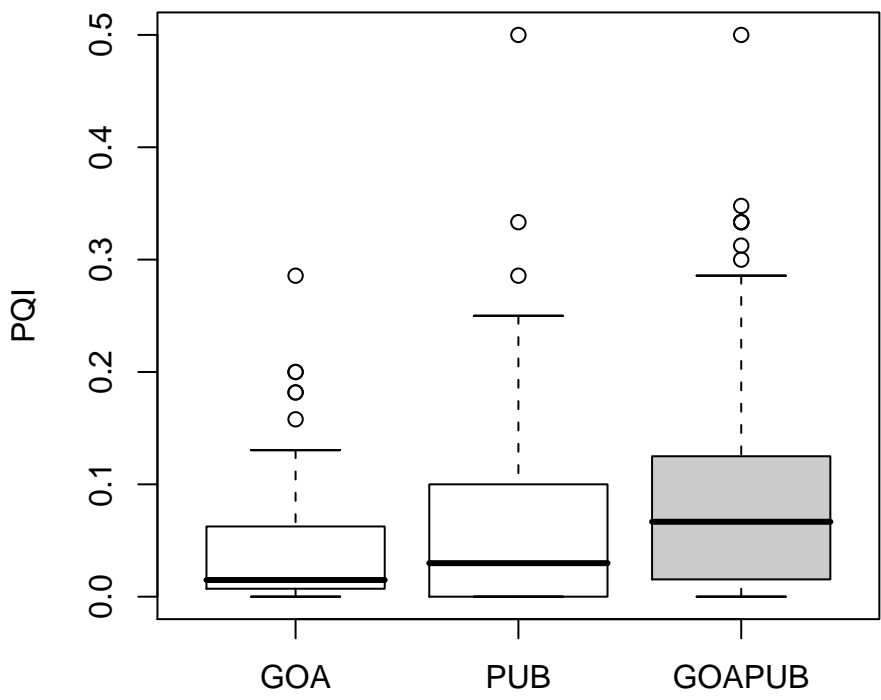

**hyperdiploid (B)**

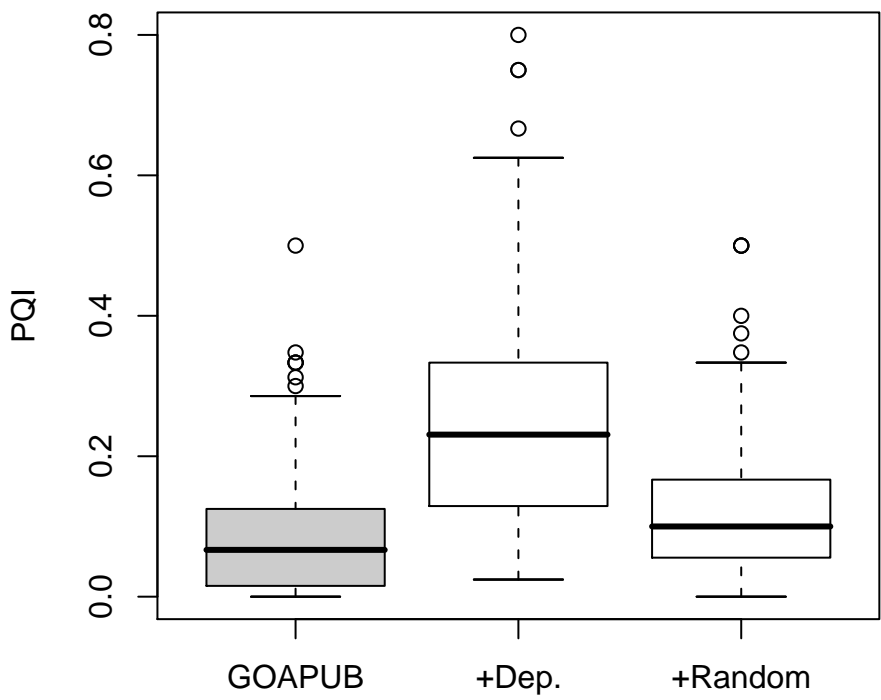

**novel (A)**

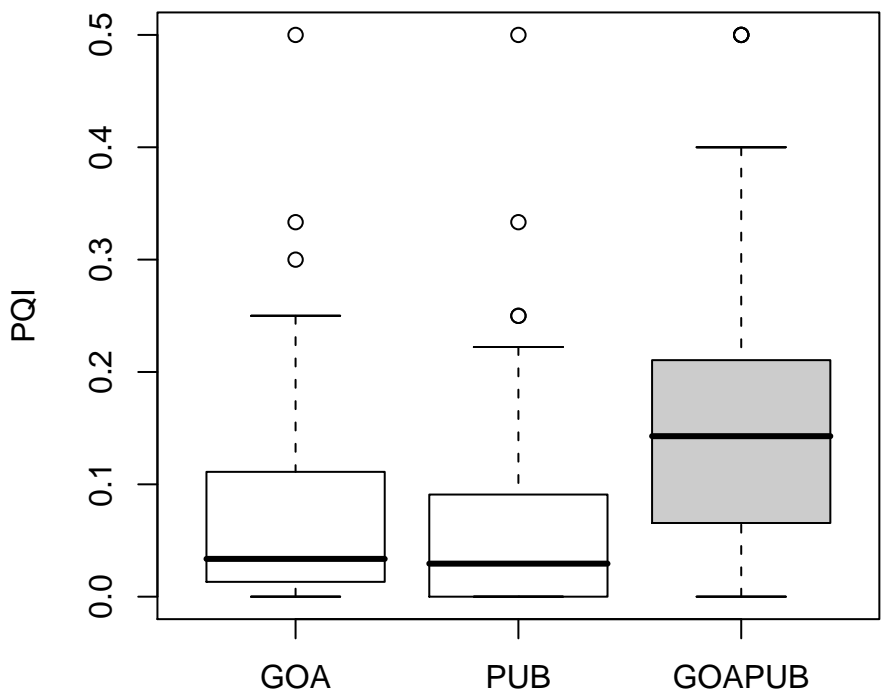

**novel (B)**

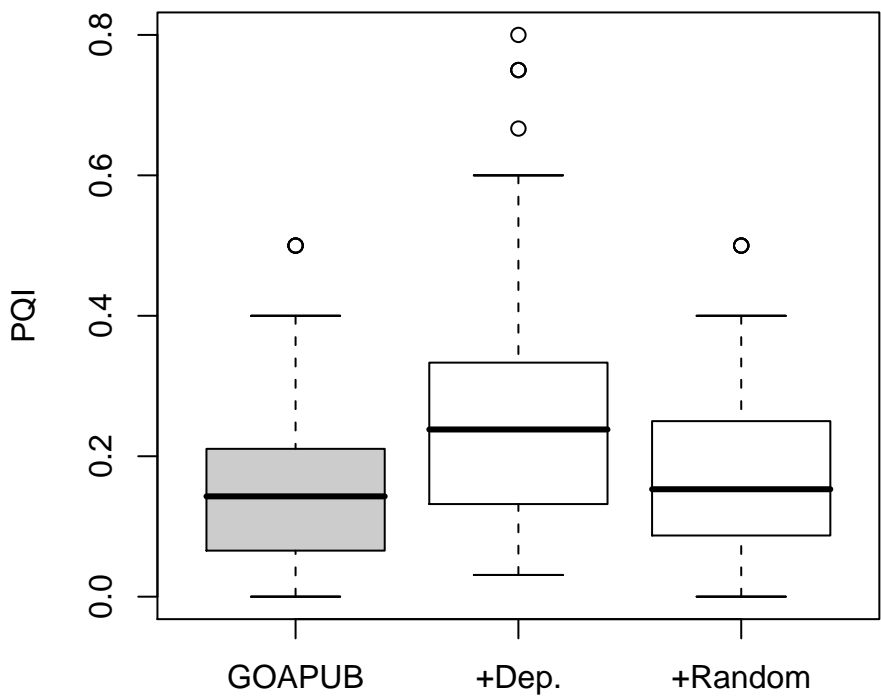

**tel-aml1 (A)**

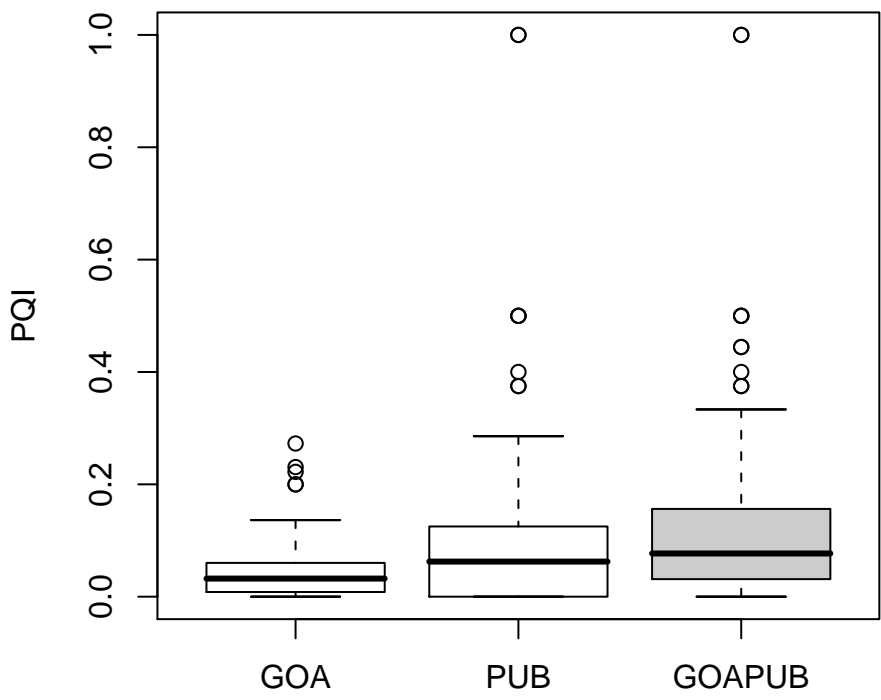

**tel-aml1 (B)**

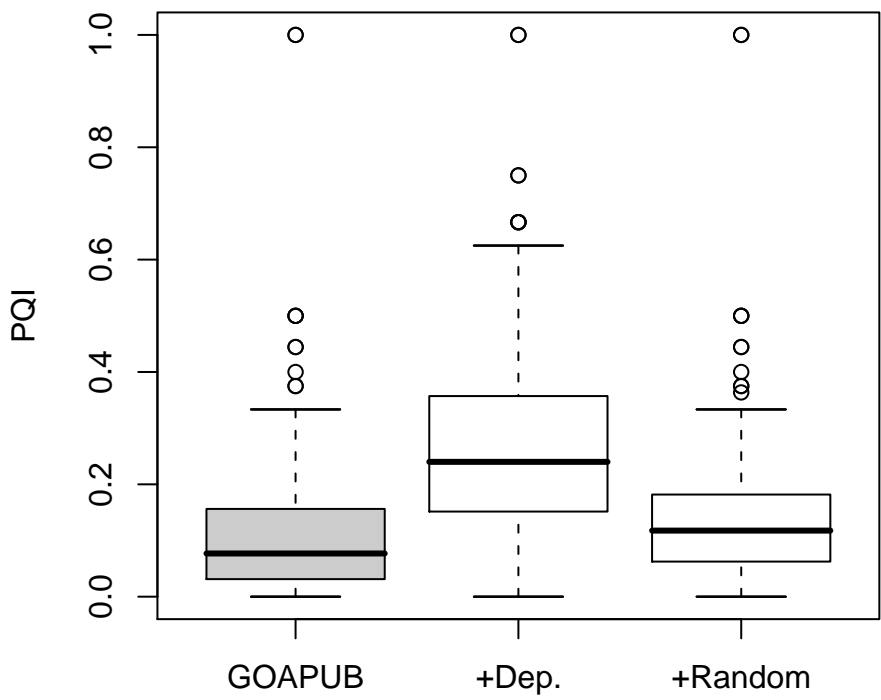

Supplement: Additional File 4 — Acute Lymphocyte Leukemias (ALL) clusters. (A) Boxplots of the PQIs for the Evidence (GOA), Literature (PUB) and combination of both (GOA-PUB). (B) Boxplots of the PQIs for the evidence and literature terms (GOAPUB), for the same set enriched with the associative relations (+Dep.) or a random term set of the same size (+Random). [file 1471-2105-7-241-S4.pdf]

**circadian (A)**

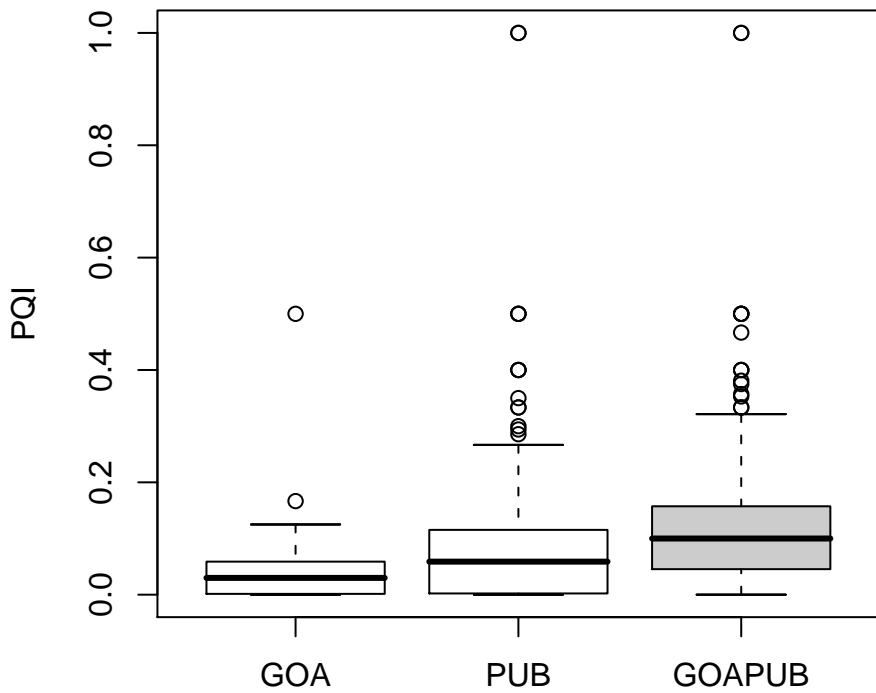

**circadian (B)**

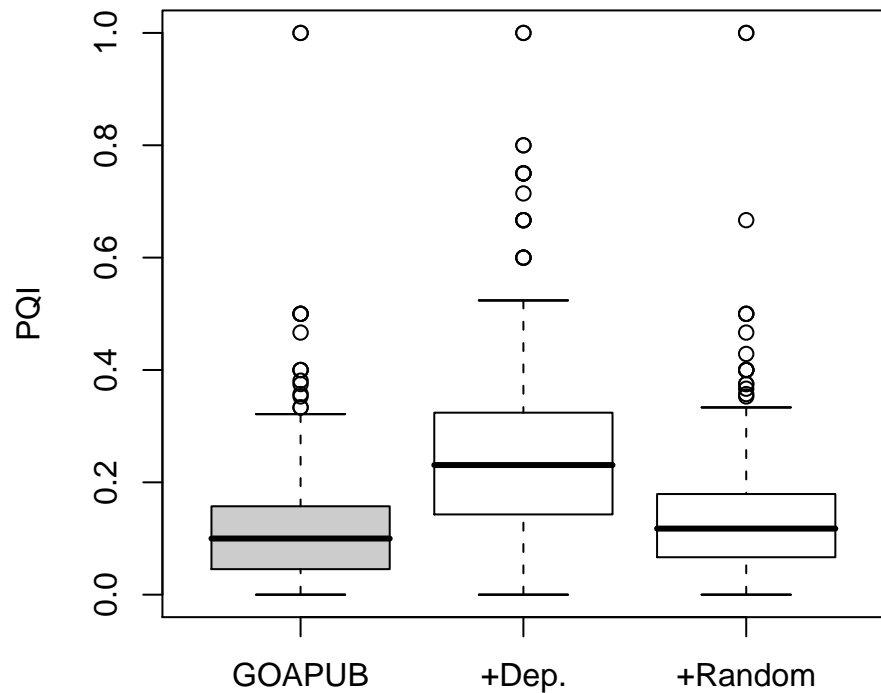

Supplement: Additional File 5 — Circadian cluster. (A) Boxplots of the PQIs for the Evidence (GOA), Literature (PUB) and combination of both (GOAPUB). (B) Boxplots of the PQIs for the evidence and literature terms (GOAPUB), for the same set enriched with the associative relations (+Dep.) or a random term set of the same size (+Random). [file 1471-2105-7-241-S5.pdf]

**lung (A)**

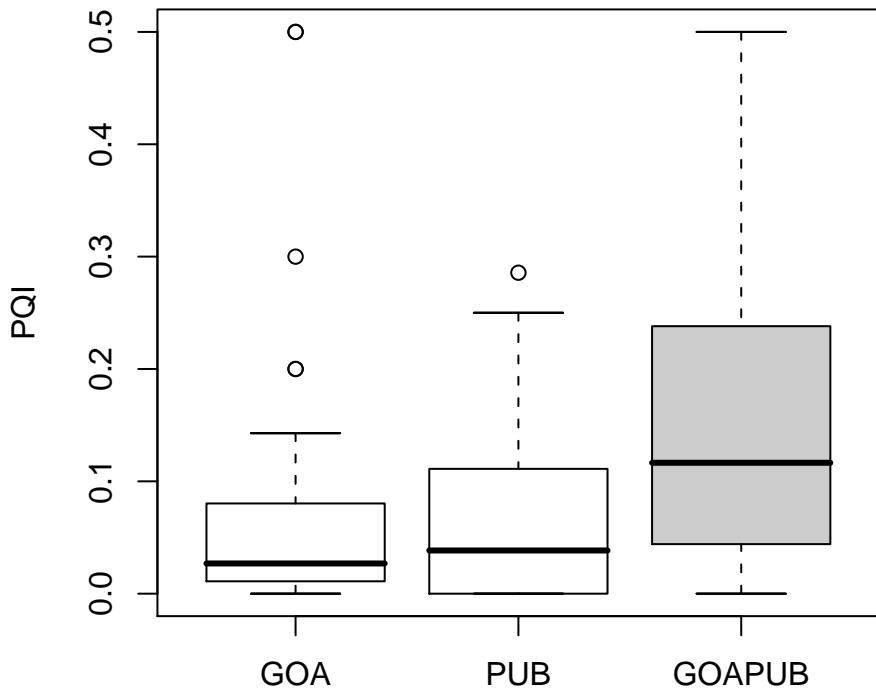

**lung (B)**

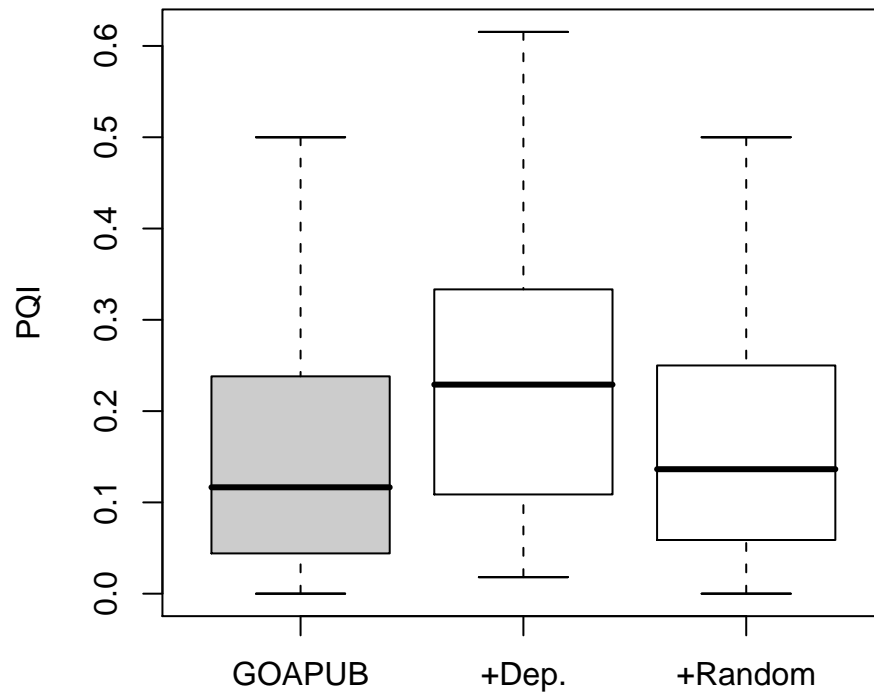

Supplement: Additional File 6 — Lung cluster. (A) Boxplots of the PQIs for the Evidence (GOA), Literature (PUB) and combination of both (GOAPUB). (B) Boxplots of the PQIs for the evidence and literature terms (GOAPUB), for the same set enriched with the associative relations (+Dep.) or a random term set of the same size (+Random). [file 1471-2105-7-241-S6.pdf]

**arpe19 (A)**

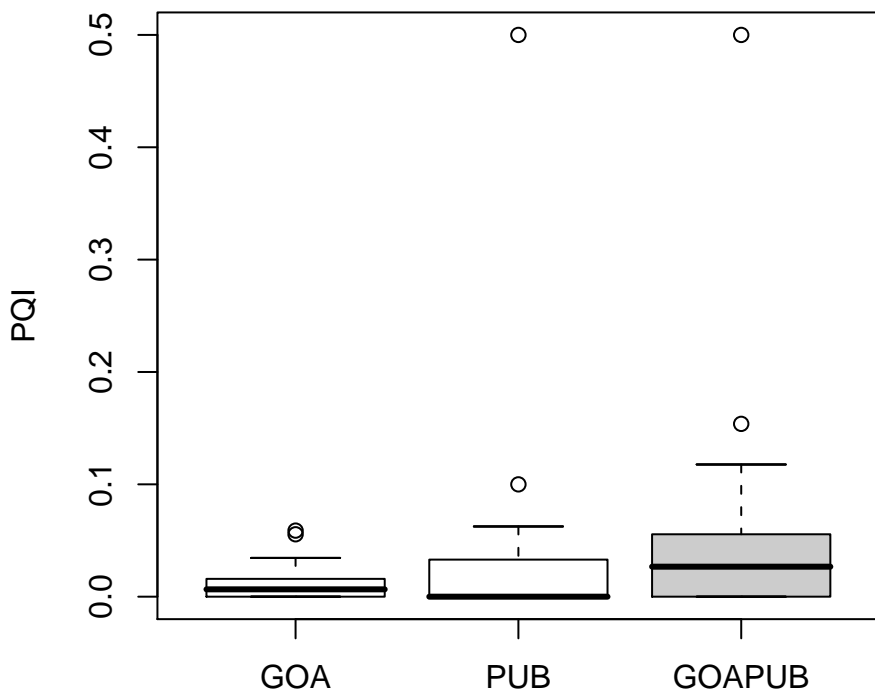

**arpe19 (B)**

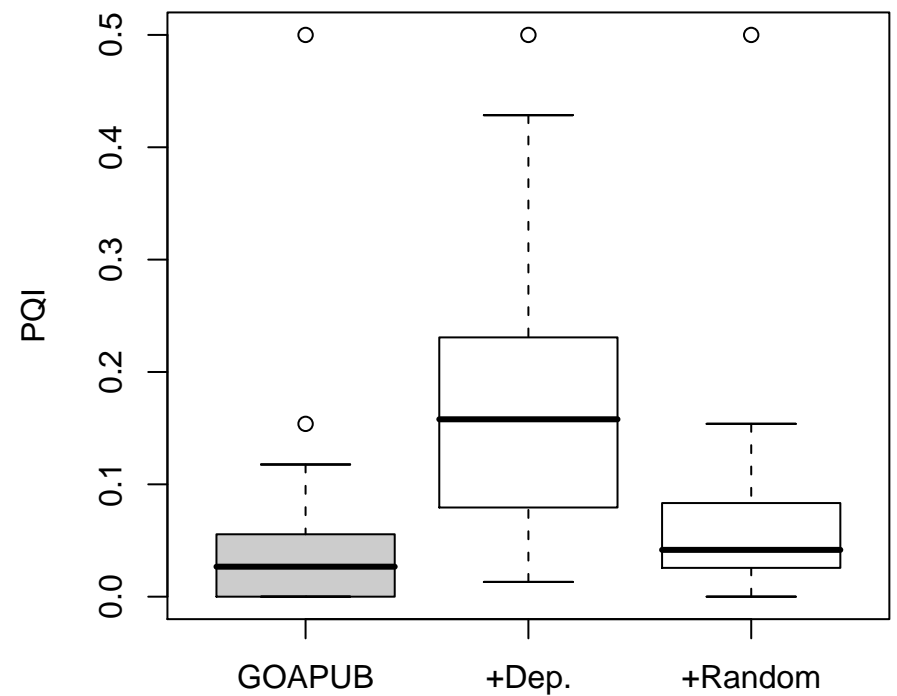

**rpe (A)**

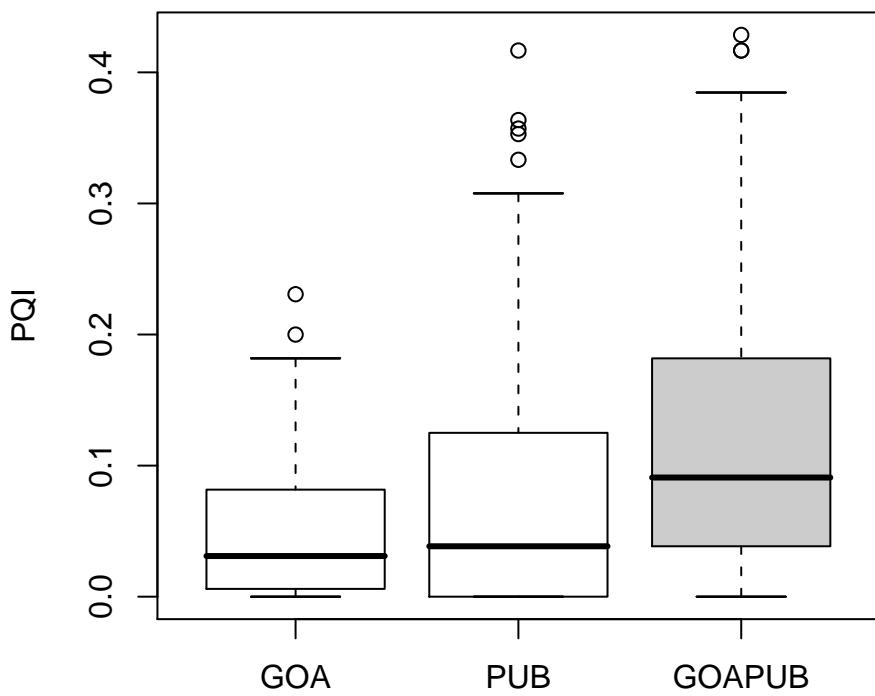

**rpe (B)**

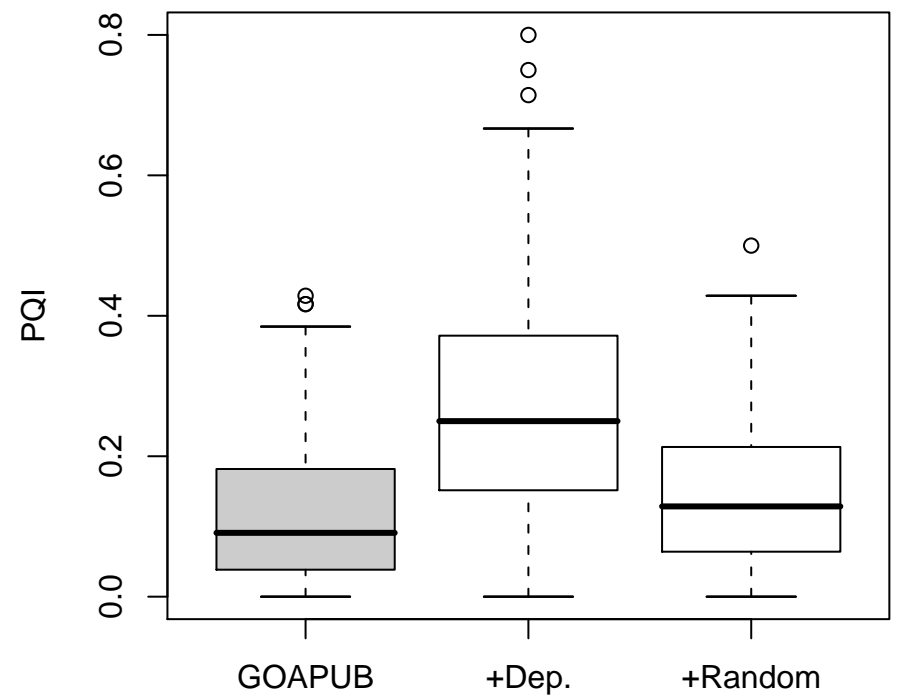

Supplement: Additional File 7 — Retina clusters. (A) Boxplots of the PQIs for the Evidence (GOA), Literature (PUB) and combination of both (GOAPUB). (B) Boxplots of the PQIs for the evidence and literature terms (GOAPUB), for the same set enriched with the associative relations (+Dep.) or a random term set of the same size (+Random). [file 1471-2105-7-241-S7.pdf]

**Alzheimer (A)**

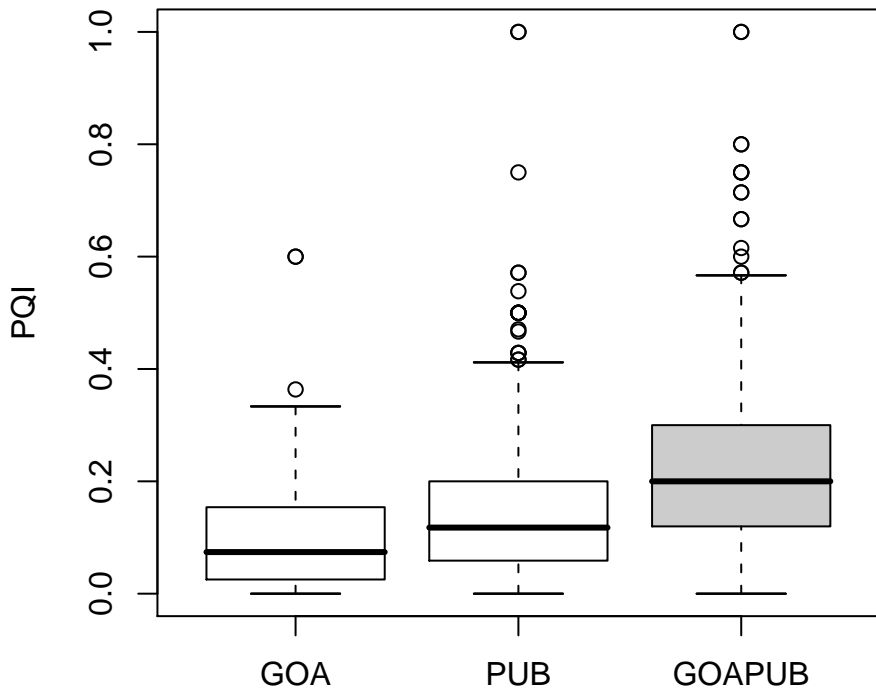

**Alzheimer (B)**

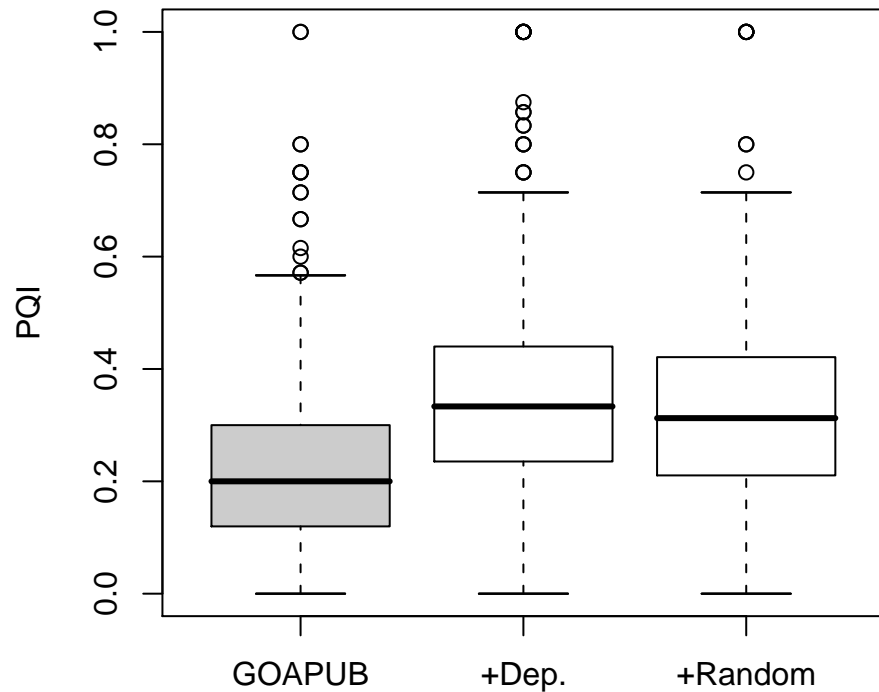

Supplement: Additional File 8 — Alzheimer's disease cluster. (A) Boxplots of the PQIs for the Evidence (GOA), Literature (PUB) and combination of both (GOAPUB). (B) Boxplots of the PQIs for the evidence and literature terms (GOAPUB), for the same set enriched with the associative relations (+Dep.) or a random term set of the same size (+Random). [file 1471-2105-7-241-S8.pdf]
