# Supplementary material for: Combining evidence, biomedical literature and statistical dependence: new insights for functional annotation of gene sets
Source: BMC Bioinformatics. 2006 May 4;7:241. doi: 10.1186/1471-2105-7-241 (PMC1482722; doi:10.1186/1471-2105-7-241)
Supplement: Additional File 9 — Enriched GO Process terms (p < = 0.05) associated with at least 4 genes in the glioGBM cluster. Color: red = terms found in GOA and PubGene, green = terms only found in GOA, blue = terms only found in PubGene. Shape: rectangle = significantly enriched annotated terms (p < = 0.05) ; ellipse = non-significantly enri-ched annotated terms (p > 0.05). [file 1471-2105-7-241-S9.pdf]

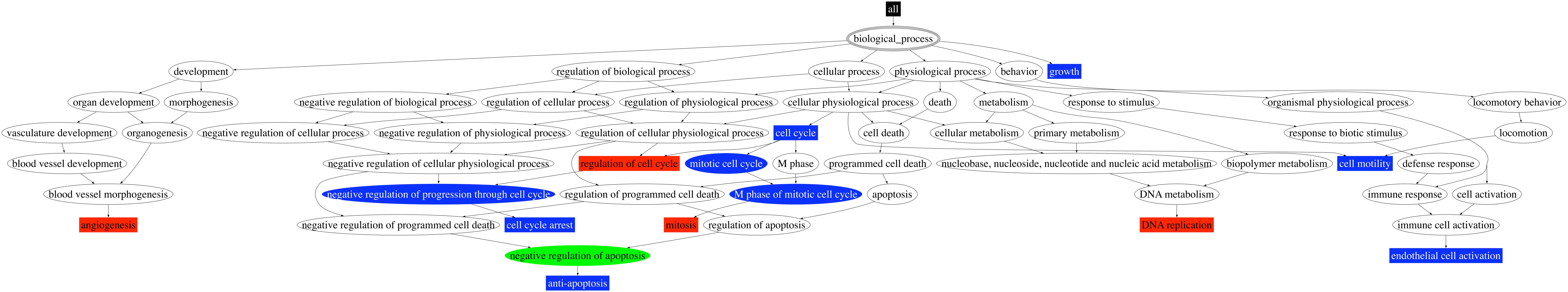
all
biological_process
development
regulation of biological process
cellular process
physiological process
behavior
growth
organ development
morphogenesis
negative regulation of biological process
regulation of cellular process
regulation of physiological process
cellular physiological process
death
metabolism
response to stimulus
organismal physiological process
locomotory behavior
vasculature development
organogenesis
negative regulation of cellular process
negative regulation of physiological process
regulation of cellular physiological process
cell cycle
cell death
cellular metabolism
primary metabolism
response to biotic stimulus
locomotion
blood vessel development
negative regulation of cellular physiological process
regulation of cell cycle
mitotic cell cycle
M phase
programmed cell death
nucleobase, nucleoside, nucleotide and nucleic acid metabolism
biopolymer metabolism
cell motility
defense response
blood vessel morphogenesis
negative regulation of progression through cell cycle
regulation of programmed cell death
M phase of mitotic cell cycle
apoptosis
DNA metabolism
immune response
cell activation
angiogenesis
negative regulation of programmed cell death
cell cycle arrest
mitosis
regulation of apoptosis
DNA replication
immune cell activation
negative regulation of apoptosis
endothelial cell activation
anti-apoptosis
