# Supplementary material for: Combining evidence, biomedical literature and statistical dependence: new insights for functional annotation of gene sets
Source: BMC Bioinformatics. 2006 May 4;7:241. doi: 10.1186/1471-2105-7-241 (PMC1482722; doi:10.1186/1471-2105-7-241)
Supplement: Additional File 10 — Enriched GO Process terms associated with at least 4 genes in the bcr-abl cluster. Color: red = terms found in GOA and PubGene, green = terms only found in GOA, blue = terms only found in PubGene. Shape: rectangle = significantly enriched annotated terms (p < = 0.05) ; ellipse = non-significantly enriched annotated terms (p > 0.05). [file 1471-2105-7-241-S10.pdf]

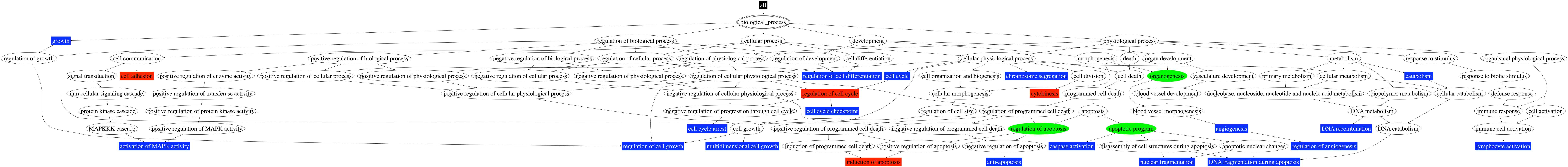

all
biological_process
growth
regulation of biological process
cellular process
development
physiological process
regulation of growth
cell communication
positive regulation of biological process
negative regulation of biological process
regulation of cellular process
regulation of physiological process
regulation of development
cell differentiation
cellular physiological process
morphogenesis
death
organ development
metabolism
response to stimulus
organismal physiological process
signal transduction
cell adhesion
positive regulation of enzyme activity
positive regulation of cellular process
positive regulation of physiological process
negative regulation of cellular process
negative regulation of physiological process
regulation of cellular physiological process
regulation of cell differentiation
cell cycle
cell organization and biogenesis
chromosome segregation
cell division
cell death
organogenesis
vasculature development
primary metabolism
cellular metabolism
catabolism
response to biotic stimulus
intracellular signaling cascade
positive regulation of transferase activity
positive regulation of cellular physiological process
negative regulation of cellular physiological process
regulation of cell cycle
cellular morphogenesis
cytokinesis
programmed cell death
blood vessel development
nucleobase, nucleoside, nucleotide and nucleic acid metabolism
biopolymer metabolism
cellular catabolism
defense response
protein kinase cascade
positive regulation of protein kinase activity
negative regulation of progression through cell cycle
cell cycle checkpoint
regulation of cell size
regulation of programmed cell death
apoptosis
blood vessel morphogenesis
DNA metabolism
immune response
cell activation
MAPKKK cascade
positive regulation of MAPK activity
cell cycle arrest
cell growth
positive regulation of programmed cell death
negative regulation of programmed cell death
regulation of apoptosis
apoptotic program
angiogenesis
DNA recombination
DNA catabolism
immune cell activation
activation of MAPK activity
regulation of cell growth
multidimensional cell growth
induction of programmed cell death
positive regulation of apoptosis
negative regulation of apoptosis
caspase activation
disassembly of cell structures during apoptosis
apoptotic nuclear changes
regulation of angiogenesis
lymphocyte activation
induction of apoptosis
anti-apoptosis
nuclear fragmentation
DNA fragmentation during apoptosis
